# Restoring and managing low-severity fire in dry-forest landscapes of the western USA

**DOI:** 10.1371/journal.pone.0172288

**Published:** 2017-02-15

**Authors:** William L. Baker

**Affiliations:** Program in Ecology/Department of Geography, Dept. 3371, 1000 E. University Ave., University of Wyoming, Laramie, Wyoming, United States of America; Chinese Academy of Forestry, CHINA

## Abstract

Low-severity fires that killed few canopy trees played a significant historical role in dry forests of the western USA and warrant restoration and management, but historical rates of burning remain uncertain. Past reconstructions focused on on dating fire years, not measuring historical rates of burning. Past statistics, including mean composite fire interval (mean CFI) and individual-tree fire interval (mean ITFI) have biases and inaccuracies if used as estimators of rates. In this study, I used regression, with a calibration dataset of 96 cases, to test whether these statistics could accurately predict two equivalent historical rates, population mean fire interval (PMFI) and fire rotation (FR). The best model, using Weibull mean ITFI, had low prediction error and *R*^2^_adj_ = 0.972. I used this model to predict historical PMFI/FR at 252 sites spanning dry forests. Historical PMFI/FR for a pool of 342 calibration and predicted sites had a mean of 39 years and median of 30 years. Short (< 25 years) mean PMFI/FRs were in Arizona and New Mexico and scattered in other states. Long (> 55 years) mean PMFI/FRs were mainly from northern New Mexico to South Dakota. Mountain sites often had a large range in PMFI/FR. Nearly all 342 estimates are for old forests with a history of primarily low-severity fire, found across only about 34% of historical dry-forest area. Frequent fire (PMFI/FR < 25 years) was found across only about 14% of historical dry-forest area, with 86% having multidecadal rates of low-severity fire. Historical fuels (e.g., understory shrubs and small trees) could fully recover between multidecadal fires, allowing some denser forests and some ecosystem processes and wildlife habitat to be less limited by fire. Lower historical rates mean less restoration treatment is needed before beginning managed fire for resource benefits, where feasible. Mimicking patterns of variability in historical low-severity fire regimes would likely benefit biological diversity and ecosystem functioning.

## Introduction

Low-severity wildfires significantly shaped dry forests in the western USA, but historical rates (e.g., mean interval, area burned) of these fires remain uncertain in a time of altered and further changing fire regimes. Low-severity fires periodically burned the understory of historical dry forests, changing fuel loads, composition, diversity, and ecosystem processes without killing most canopy trees [1–2]. Dry forests in the western USA cover 25.5 million ha and include dry pine forests, dominated by ponderosa pine (*Pinus ponderosa*) or other dry pines, and dry mixed-conifer forests that also have firs (*Abies concolor*, *A*. *grandis*, *Pseudotsuga menziesii)* and other trees [3]. Past reconstructions of low-severity fire in dry forests, using tree-rings, focused on long records of dated fire years in small plots, and most were not intended to accurately estimate key rate parameters of low-severity fire [1–2] needed to restore and manage low-severity fire across large landscapes. These small-plot reconstructions have known inaccuracies and biases if inappropriately used for this purpose [1, 4–11]. Fortunately, new landscape-scale and small-plot reconstruction methods [1, 11] overcome many known inaccuracies and biases in estimating historical low-severity fire rates, but limited new estimates are available.

This situation leaves a weak current basis for restoring and managing low-severity fire, using historical rates as a guide, across dry-forest landscapes. Here I: (1) develop regressions for estimating mean historical rates of low-severity fire from past reconstructions using a calibration dataset, and then (2) apply these regressions to estimate mean historical rates of low-severity fire for a large dataset of past reconstructions across the western USA, and (3) assess the applicability of these new estimates across dry-forest landscapes. These new estimates are directly usable in restoring and managing low-severity fire in the parts of dry forests of the western USA where low-severity fire was historically predominant, and provide a West-wide perspective on variability in historical mean rates of low-severity fire in these parts of dry forests. As discussed later, variability around mean rates is also an essential attribute of a low-severity fire regime.

Estimated mean historical rates of low-severity fire need to be fairly accurate, for restoring and managing low-severity fire, because key effects of fires on biological diversity, ecosystem functioning, and post-fire recovery operate significantly differently across a narrow range of mean rates. For example, understory fuels in dry forests, reduced by a single fire, often recover to pre-fire levels in about 7–25 years [12–14]. If mean fire intervals for low-severity fires were 10–15 years, understory fuels would often have been kept at relatively low levels, but if mean intervals were 25 years or more, then understory fuels would more often have been fully recovered and generally higher. Fires that are too frequent can reduce the ecological roles of the forest floor in replenishing soil nutrients and organic matter, enhancing absorption of water and nutrients, and providing habitat for microbial communities, potentially reducing long-term forest productivity [15]. Habitat for wildlife that use snags or down wood could be adversely affected by fire that is too frequent [15], which can also reduce understory plant species richness, possibly due to depletion of soil nitrogen [16]. Native shrubs, historically abundant in some dry forests, may also be reduced by fire at intervals less than about 20–30 years [17]. However, fire-stimulated shrubs in the understory of dry forests may also decline if low-severity fire rates are too low [18]. Insufficient low-severity fire can allow tree density or other understory shrubs to increase, reducing nutrient cycling and understory diversity, and increasing fire severity [16, 19].

Maintenance of tree populations in dry forests also depends on the balance between tree natality and mortality, a balance strongly shaped by rates and patterns of fires. Fire intervals for successful tree regeneration were likely long relative to historical mean intervals, as fires at short intervals can kill most small trees [6]. Patchy surface fires could alone allow survival of small trees in unburned areas [20]. Also, seedlings regenerating in openings may produce limited fuels, enhancing fire patchiness that favors seedling survival [21]. Where fire kills overstory trees, a resulting mineral seedbed and reduced competition with grass can enhance tree regeneration, if other factors (e.g., seed production) co-occur [22]. A fire-quiescent period is also needed [23]. Long intervals may occur over large areas in wet periods, or stochastically from variability in fire. In contrast, mortality of larger trees from single low-severity fires can reach 7–8%; if repeated every 10 years, larger trees could be reduced by half in a century, but, assuming the same 7–8% rate repeated every 50 years, larger trees would be halved in 500 years [17]. Thus, tree populations, both young and old trees, are sensitive to rates and patterns of low-severity fire.

Rates and patterns of low-severity fire also affect how resistant and resilient dry forests are to future fire, drought, and beetle outbreaks [24]. Open, low-density forests relatively free of shrubs and small trees can be produced by repeated low-severity fires, and may be more resistant to subsequent higher-intensity fires than are denser forests, with more shrubs and small trees [25]. Forests subject to repeated low-severity fires could even be self-limiting, if the rate of fires is high, possibly promoting continuing low-severity fire rather than higher-severity fires [26]. However, if a deficiency in tree regeneration occurs because of too-frequent fires, dry forests would be vulnerable to subsequent regeneration lags or failures after droughts and beetle outbreaks that are a higher current risk than are severe fires [24]. Too little low-severity fire could increase fire severity, but too much could reduce higher-severity fires that enhance spatial heterogeneity, a key source of forest resilience to future disturbances [3].

Research has enhanced understanding of the importance of rates and patterns of low-severity fire to biological diversity, ecosystem functioning, and sustainability of dry forests, but estimated historical rates and patterns of low-severity fire remain uncertain. Newer methods for accurately reconstructing rates of historical low-severity fire promise to eventually resolve uncertainty, but improved estimates, the focus here, might be possible from past research.

## Measures and estimators of mean rates of low-severity fires

### Terms and measures

A *low-severity fire* in this study is a fire that burns in the understory of a forest, and is often defined as causing mortality or topkill of no more than about 20% of stand basal area [27–28]. These fires are not usually burning in the canopy independently, instead torching upwards from surface fuels into single or small groups of trees. These fires could also be called low-moderate severity to reflect some canopy mortality, but the extent of canopy mortality from these fires is poorly known [17].

Several measures of mean rates of fire also need explanation. At a point in a landscape, the average interval between fires is the point *mean fire interval* (point MFI). The average MFI across multiple points in a landscape provides a sample estimate of the *population mean fire interval* (PMFI) for a particular landscape, which is the grand mean fire interval across the landscape [6]. Fire-interval data at points have interval distributions that often are skewed, not normally distributed. Alternative measures of central tendency, such as the median, can characterize these distributions. These distributions often can also be fit by the flexible two- or three-parameter Weibull distribution, which has a shape parameter that describes the form of the distribution (e.g., lognormal), a scale parameter that represents the 63^rd^ percentile of the distribution, and a shift parameter to set the location of the distribution [29]. The mean and median of the fitted Weibull distribution, which can offset unusual values in actual data [29], are useful alternative measures of central tendency. Descriptors of variation (e.g., standard deviation) are relevant for all measures. The *fire rotation* (FR) is the expected time for fire to burn an area equal to the area of a landscape of interest [17]. The FR for a landscape is equivalent to the PMFI, which was shown analytically [6] and through simulation [7–8]. Fire-interval data at points can be used to estimate the PMFI, or area-burned data across a landscape can be used to estimate the FR. PMFI estimates at points and FR estimates across areas are the fundamental, equivalent estimators of mean rates of fire, as they show how often points experience fire and the equivalent time it takes for fire to burn across a landscape.

### Estimators of the Population Mean Fire Interval (PMFI)

For reconstructions of mean low-severity fire rates in the pre-EuroAmerican period, which are predominantly derived using tree-ring and fire-scar methods, the actual intervals needed for estimating PMFI can be sampled and processed in several ways. Fires do not physically leave a scar on every tree that burns [30], and the scarring fraction (SF), the fraction of live trees that receive a scar from a fire, may be moderate or even low. The intervals derived from scarred trees are thus simply estimators of the actual fire intervals that occurred at a point.

The most widely used fire-interval estimator is the mean composite fire interval (mean CFI), often also called the mean fire-return interval (MFRI) or even, to confuse matters, the MFI itself, which is not the estimator but instead what is being estimated. This estimator seeks to offset the fact that SF is < 1.0 by compositing scar records across a set of nearby trees, which together are expected to contain a more complete record of fires that burned the point. To calculate mean CFI, the user creates a pooled “composite” list of fire years that burned any tree in a set of sample trees, then the estimated intervals are those between fire years in the composite list. However, this composite list of all fires may contain small spot fires that have little ecological effect, and users often also report estimates for larger fires that scarred more than 10%, 25%, or another percentage of scarred trees. Various measures of central tendency can be calculated, including the mean, median, and Weibull measures. I distinguish variants here using combined terms, such as mean CFI-all fires, mean CFI-10% scarred, or median CFI-25% scarred. Mean CFI-10% scarred, for example, is the mean composite fire interval for fires recorded on ≥ 10% of scarred trees.

Another commonly used estimator is the mean individual-tree fire interval (mean ITFI). This estimator is calculated in two steps. First, the intervals between fires on an individual scarred tree are used to estimate the MFI for that tree. Second, the grand mean of each tree’s estimated MFI is calculated across a set of sample trees. In this case, restrictions (e.g., 25% scarred) are not used, but alternative measures of central tendency are, so there are fewer variants.

Finally, we developed an estimator, the mean all-tree fire interval (mean ATFI), which seeks to offset SF < 1.0 by using an estimated SF to predict the total number of scars that would have occurred if SF was 1.0 [7–8, 11]. This estimator has been shown to be the best available estimator of PMFI [11], but it is not used in this paper because few ATFI estimates are currently available.

### Estimators of the Fire Rotation (FR)

Area-burned estimates for calculating FR can be derived from three main sources: (1) area burned in recent fires from agency polygon fire records or fire-atlas records or from remotely sensed data, (2) historical area burned from fire-year maps reconstructed from scarred-tree or plot locations, or (3) historical area burned reconstructed using a ratio method and scarred-trees or plot records, or comparable data in a table or graph.

Polygon fire records or fire-atlas records are available from public land-management agencies, and are most complete and accurate after about A.D. 1980. Early data are often from fire perimeters sketched on a map, but later data may have been from remotely-sensed data [31]. Small fires were not always mapped. Accuracy of boundaries of fires in fire-atlas data, relative to tree-ring reconstructions and remote-sensing data, was moderately high in one study, sufficiently accurate to use in some research [31]. In another study, tree-ring methods underestimated fire extent relative to fire-atlas maps, which also had some errors [32]. A larger study showed closer agreement between fire-atlas data and tree-ring reconstructions of fires [1].

Fire-year maps are typically reconstructed from tree-ring and fire-scar data collected at a grid of points or a set of random points. Fire scars near the points are dated, dates are displayed on a map or in GIS, and a fire perimeter is placed around the points common to a fire year [33–34]. The boundary is positioned using a set of fire-spread principles [35], Voronoi polygons centered on the points [1], convex hulls [32], fuzzy-set methods [36], inverse-distance weighting [23, 33], or indicator-kriging [33–34]. If grid points are close, unburned area may be most accurately mapped, but a larger grid spacing is often needed to allow sufficient area to be sampled, leading to less precision in boundaries and unburned areas [34]. Smaller fires also will be missed more often with larger grid spacing. Larger fires that contribute most to fire rotation are mapped the best. Fire rotation has been shown to be estimated within about 10% of the value obtained from fire-atlas data [1, 11].

A non-spatial ratio method estimates area burned within a study area as proportional to the percentage of sample trees scarred in a particular fire year or the percentage of plots in which a particular fire year is recorded on sample trees. The equation [37] is:

$$A_{i}=(AT*NS_{i})/(NST-NRE)$$ (1)

where *A*_*i*_ is area burned in year *i*, *AT* is the study area size, *NS*_*i*_ is the number of scarred trees or plots recording a fire in year *i*, *NST* is the total number of scarred trees or plots, and *NRE* is the number of scarred trees or plots eliminated by subsequent fires. This method is most accurate when the number of scarred trees or plots is large and these are well distributed across a sample area [1, 37]. However, scarred trees are often clustered [30], which could lead to ratio estimates that are biased and too short. Because the location of scarred trees or plots is not used, unburned area may also be underestimated. In a large modern corroboration study, the ratio method accurately estimated area burned of larger fires (> 100 ha), that accounted for 97% of total area burned, and fire rotation from total plots was 89% of fire rotation from fire-atlas data [1].

FR can be calculated, using any of the three sources of data, by the equation [17]:

FR=(ObservationPeriod/FractionBurned) (2)

where *FR* is fire rotation, in years, *ObservationPeriod* is the period, in years, for which there are mapped or reconstructed records of fire, and *FractionBurned* is the fraction of the study area estimated to have burned during the observation period, obtained by summing the areas of fires or the estimated fraction burned from ratio estimates.

### Perspectives on estimating PMFI/FR and interpreting mean CFI

A central area of analysis and discussion by our research group has been about whether past mean CFI and ITFI estimates from small plots accurately estimate PMFI/FR. Other studies (e.g. [38]) were more focused on reconstructing a long history of dated fire years across a network of locations, not so much accurate rates of fire across landscapes. I continue the rate focus here. An earlier review suggested mean CFI is too short and mean ITFI is too long as an estimator of PMFI/FR [6]. This study suggested mean CFI was often too short from compositing across too much area or samples and mean ITFI was too long, as it does not offset unrecorded fires that occur because SF is < 1.0 [6].

Reflecting a need for rate estimates, some studies mostly used mean CFI as comparable to, or effectively an estimator of FR [39–40]. Others also used historical median CFI as an estimator of historical FR [41]. Another compared estimated median CFI, ITFI, and FR, found median ITFI was closest to FR, and suggested median ITFI might be used to estimate FR in low-severity fire regimes [42]. In contrast, other studies suggested fire scars provide estimates of the PMFI/FR that are generally too long: “… our findings clearly demonstrate that analysis of fire scars will likely underestimate past fire occurrence” ([10]:1500). However, when compositing fire-scar records over larger areas and more trees, mean CFI declines toward 1.0, a fire every year [1, 43], an estimate of PMFI/FR that is nearly always too short. Given uncertainty about estimators of low-severity fire rates, some studies suggested that summary statistics, such as mean CFI or FR, should not even be used in restoring and managing low-severity fire (e.g. [44]).

Other studies suggested that multiple descriptors of fire regimes (i.e., including mean CFI) are desirable (e.g. [1]). Studies, that favored mean CFI and ITFI as one of multiple statistics, suggested they must be interpreted correctly. For example, regarding mean CFI-all fires, one study said it was not designed to estimate area burned, and if it does not, that is not a problem in mean CFI, but an error in interpreting it [1]. Other studies also suggested it is a problem if mean CFIs are interpreted as indicating how often the entire stand burned “… since fires are quite variable in burn patterns” ([2]:1091). Similarly, other studies suggested managers need to recognize that fires indicated by mean CFI burned in variable spatial and temporal patterns, including unburned areas [45]. A study in California said: “… the composite MFIs are not equivalent to average point fire intervals, population means [sic] fire intervals or natural fire rotation. They are an estimation of average intervals between fires of any size, or of an estimated size class, occurring anywhere within a study area” ([46]:52). That mean CFI declines with increasing sampling area is also interpreted by some not as a fundamental flaw [6], but instead as an added descriptor of a fire regime [47–49]. Complex power-function patterns across spatial scales, observed as mean CFI declines toward 1.0 with more samples, are thought in this study to elucidate cross-scale spatial properties of fire regimes. Thus, “… measures of fire frequency are area dependent, and … fire return intervals cannot be described by a single number independent of spatial scale” ([48]:820). However, scale-dependent values are only known for CFI measures, not other rate measures. In summary, there is now general agreement that mean CFI and its variants (e.g., median CFI) and ITFI are not intended to estimate the PMFI/FR. Mean CFI is accepted to not indicate area burned, the pattern of the fire, or PMFI/FR.

Accurate estimators of the PMFI/FR are still needed. Fortunately, recent modern calibrations have validated new methods for estimating PMFI and FR that do not need to use mean CFI or ITFI and have promising accuracy [1, 11]. However, it may be decades before better estimates from these new methods become sufficiently common to be able to guide restoration and management of low-severity fire. In the meantime, past mean CFI and ITFI plot estimates are abundant, and required large efforts to gather and process. Moreover, plot data on fire history likely will remain a fundamental sampling component of spatial fire histories, and could provide detail about spatial variability in FR and MFI across landscapes. Mean ITFI is less studied; it remains unclear how it might perform as an estimator of PMFI/FR, but it may suffer from the unrecorded fire problem, so that mean ITFI may be too long [6]. Now that there are more spatial estimates of FR, further analysis of the relationships of CFI, ITFI, and PMFI/FR is warranted, to see whether a variant of CFI or ITFI may estimate PMFI/FR.

## Materials and methods

**Fig 1:**
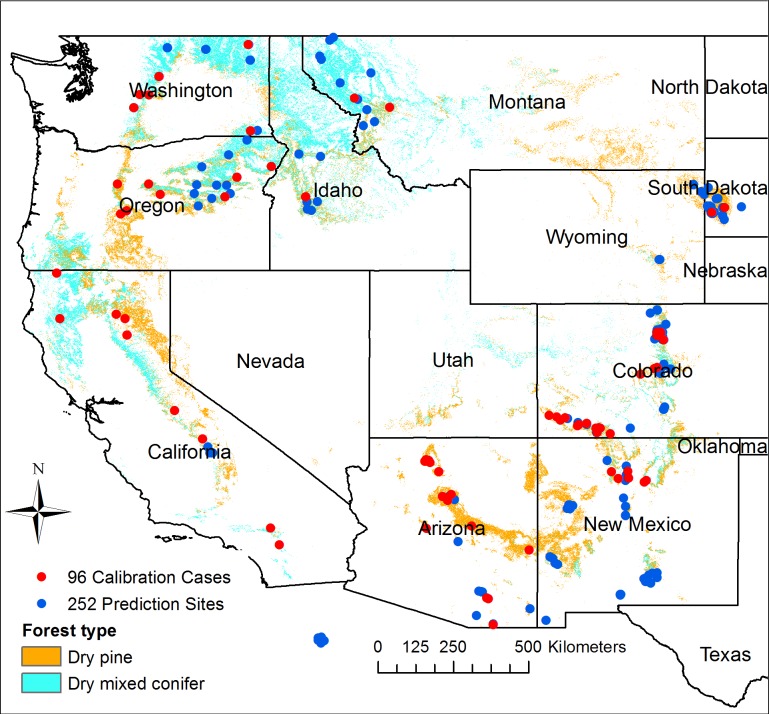
The 96 calibration cases and 252 prediction sites from the International Multiproxy Paleofire Database. Note that multiple plots were often done near one site, thus the number of dots is fewer.

I assembled two datasets for analyzing the relationships of CFI, ITFI, and PMFI/FR in dry forests of the western USA (Fig 1) using an analysis of bias and inaccuracy followed by regression analysis. I also recorded and analyzed fire-history sampling measures (e.g., number of samples) and their effects on these relationships.

### The 252-site fire-history dataset

To obtain a large sample of fire-history sites in dry forests to use to analyze methods and estimators in common use, I searched the International Multiproxy Paleofire Database (IMPD) (https://www.ncdc.noaa.gov/data-access/paleoclimatology-data/datasets/fire-history) for all fire-scar sites between 102° and 125° west longitude and 30° and 60° north latitude, finding 436 sites. I excluded 77 sites not clearly in dry pine or dry mixed-conifer forests. Some were also excluded because their FHX file (containing the fire-history data) in the IMPD was not usable (n = 26), the dataset was too small (n = 6) or calculations could not be completed (n = 12). I also removed 63 sites usable in a calibration dataset, described next, which left 252 sites. I left in 9 sites from Mexico and one from Canada that are nearby and relevant to the western USA.

I downloaded from the IMPD an FHX file containing fire-history records for each site and used the Fire History Analysis and Exploration System (FHAES; frames.nbii.gov) Version 2.0.2 [50] to calculate CFI and ITFI estimators. To reduce differences in the period of record, I restricted calculations for all sites to the period extending from the earliest fire to the latest fire within the A.D. 1600–1900 period. The purpose of restricting analysis to fire-to-fire periods is that scar-to-scar fire intervals are traditionally used. I did not want to introduce a possible confounding variable by using an arbitrary period. After restriction, I omitted sites with < 50 years of record, an arbitrary criterion aimed at minimizing short records.

For each case, I also recorded ancillary information, from the original publication reporting the study or from the FHX file, including the sample area, the number of sampled scarred trees, the total number of fire scars across all sampled trees, the analysis years used. and the types of targeted sampling used, including: (1) seeking the best information/longest record, (2) seeking multi-scarred trees, (3) seeking clusters of scarred trees, (4) seeking scars on dead wood, or (5) placing plots or selecting study areas in areas with many scarred trees or in old forests with long records of fire. I also recorded whether fire severity was studied, and I recorded the location of the samples found in the FHX file or publication.

### The 96-case calibration and analysis dataset

To analyze the relationship of CFI and ITFI estimators and FR, I searched for and found 44 fire-history studies with 96 fire-history reconstructions and alternative calculations of fire rates in dry forests in which the study: (1) estimated CFIs and/or ITFIs and (2) in areas of at least 80 ha, also estimated FR or provided data sufficient to allow FR to be calculated from data in the paper or in an FHX file (S1 Table). The purpose of this dataset was to analyze whether CFI and ITFI estimators can predict FR. I included all sites from the IMPD, meeting the criteria defined earlier, for which sample area was given and was ≥ 80 ha, and for which there was a usable FHX file. Other sites > 80 ha were included that did not have an FHX file, but were documented in a publication. If area was reported as a range, I used the midpoint. The 80-ha minimum is an arbitrary limit to increase the area used for estimating FR. Analysis periods did not need to be pre-EuroAmerican or identical among sites, but had to have ≥ 50 years of record. If measures were not calculated in the study, I restricted analysis to scar-to-scar intervals, beginning with the first scar after ≥ 10 samples had accumulated, and ending with the last fire.

FR was calculated in the study, or by me if the study did not do this, using the previously-described area-burned estimates: (1) area burned from agency polygon fire records (n = 1) or fire-atlas records (n = 2), (2) estimates of area burned from fire-year maps reconstructed from scarred-tree or plot locations (n = 24), or (3) estimates of area burned from the ratio method and scarred-tree or plot locations (n = 63), or data in a table or graph (n = 6). For published studies, I recorded whether FR was estimated from total number of scarred trees/plots or recorders. In a few cases, this was uncertain and I recorded the most likely. A recorder is a tree scarred at least once, which increases the probability of recording fires [30]. If the study did not estimate FR, I used FHAES and Minitab to estimate FR from fire-history data in the IMPD for sites for which an FHX file was available and usable. I copied the summary table, provided in FHAES for each FHX file, into Minitab 17 [51] to do calculations. I made ratio estimates, and calculated them separately based on both total number of scarred trees and number of recorders. Sites were included more than once if different methods to calculate FR were provided in the study or could be calculated. As in the case of the 252-site dataset, I obtained and recorded ancillary information for each site.

### The 342-site merged dataset

To allow calculation of histograms for particular attributes across all the sites, I merged sites in the two datasets. I removed post-EuroAmerican sites from the 96-case calibration dataset, then merged it with the 252-site prediction dataset, yielding a dataset of 342 sites (S2 Table). These include some alternative estimates from the same site or area by different studies or from using different methods, data sources, time periods or with different boundaries or other differences.

I did a rough analysis of whether sampled stands were old forests in the pre-EuroAmerican era. Old-growth dry forests are generally at least 150–200 years old, but also have attributes other than age [52], so here I call forests older than 150–200 years just “old forests.” To roughly estimate the age of sampled forest stands, I used the beginning year of analysis for each stand, as defined in the study (first fire year if not). Stands with beginning years before A.D. 1700 were likely generally ≥ 200 years old in A.D. 1900, thus would have been old forests in the pre-EuroAmerican era. Although some could have been younger, if the oldest sample trees were not abundant, often the beginning year of analysis was defined by a minimum number of sample trees (e.g. [10]). Although imprecise, this should roughly estimate sampling in old forests. I also reviewed GLO-survey and aerial-photo reconstructions of fire severity to assess the percentage of historical landscapes with a history of predominantly low-severity fire. The GLO reconstructions use a calibrated and validated low-severity fire model [53]. The calibrated model predicts low-severity fire where historical tree density was < 178 trees/ha, percentage of large trees was > 29.2%, and percentage of small trees was < 46.9% [53].

### Can CFI and ITFI measures predict PMFI/FR?

The calibration dataset included 21 estimators of the rate of low-severity fires based on CFI, ITFI, and PMFI/FR and three sample-size variables. Sample-size variables included sample area (ha), total number of scarred trees, and scar density, expressed as total scarred trees per 100 ha (e.g. [54]). These variables are included because previous analyses found that CFI estimators were related to sample size [6]. The 21 estimators of the rate of low-severity fires included five measures of central tendency (mean, median, Weibull scale, Weibull mean, and Weibull median) for CFI-all fires, CFI-10% scarred, CFI-25% scarred, ITFI, plus the PMFI/FR based on recorders.

These 21 variables are used to individually predict PMFI/FR based on total scarred trees/plots, not based on recorders, for several reasons. Most of the best available estimates, from fire-year maps and ratio estimates using plots in a grid, are based on fires from total scarred trees in the plot. For ratio estimates from just scarred trees, recorders or all scarred trees each have strengths and limitations (S1 Text), summarized here. The use of all scarred trees is consistent with most plot-scale fire-year estimates. Recorders are two to three times less abundant than single-scarred trees, so area burned is inherently less detailed if only recorders are used, likely generally inflating area burned and shortening the estimated PMFI/FR. However, recorders do have a higher probability, than do unscarred trees, of recording a fire or of documenting it did not burn at a particular point [30]. Recorders are also multi-scarred trees, that inherently omit unscarred and single-scarred trees, that can indicate where fires did not burn, also inflating area burned and shortening PMFI/FR. PMFI/FR estimates from targeted trees (typically multi-scarred) were reduced to about 86–95% of estimates from equal-size probabilistic samples [55], supporting this expected effect. Also, about 1/3 of fires may be missed if only recorders are used [S1 Text]. More research is needed on using unscarred trees, single-scarred trees, recorders (≥ 2 scars), or all scarred trees to estimate area burned, but all scarred trees likely provide the best estimates.

To understand the direction and magnitude of differences between the 21 estimators and the PMFI/FR, I calculated bias and inaccuracy for the 21 estimators relative to PMFI/FR-total scarred trees/plots for the calibration dataset. Bias is quantified by relative mean error (RME):

$$RME=\sum_{i=1}^{n}\left[(M_{i}-FR_{i})/FR_{i}\right]/n$$ (3)

where *M*_*i*_ is value *i* of *n* total available estimates for CFI or ITFI estimator *M* of the 21 estimators and *FR*_*i*_ is the corresponding estimate of PMFI/FR-total scarred trees/plots [56]. RME measures relative bias as sample sizes differ. I also calculated the standard error of each mean and tested the null hypothesis that mean bias is zero using a one-sample *t*-test in Minitab 17 [51]. Inaccuracy or error was also calculated using a relative measure, relative mean absolute error (RMAE):

$$RMAE=\sum_{i=1}^{n}[|(M_{i}-FR_{i})|/FR_{i}]/n$$ (4)

where symbols are as above. This quantifies the difference or error between the 21 estimators versus PMFI/FR-total scarred trees/plots as a percentage of this PMFI/FR estimate [56]. I also calculated the standard error of each mean and then tested the null hypothesis that mean inaccuracy is zero using a one-sample *t*-test in Minitab 17 [51].

**Fig 2:**
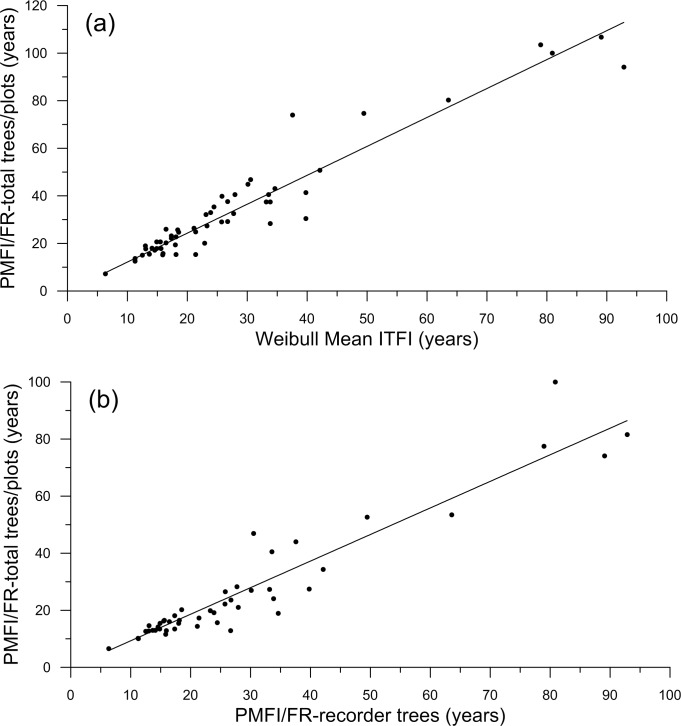
Scatterplots showing the linear relationships between: (a) Weibull mean ITFI and fire rotation-total trees/plots, and (b) Fire rotation-total trees/plots and fire rotation-recorder trees.

Can bias and inaccuracy be overcome by adjusting estimators using regression models? Scatter plots showed that PMFI/FR-total scarred trees/plots versus CFI and ITFI estimators were generally linear (e.g., Fig 2A), thus I fit linear regression models, using the lm function in R version 3.2.3 [57], to predict PMFI/FR-total scarred trees/plots from each of the 21 estimators. Sample size differed among the regressions, because individual estimators were not available for all 96 cases. After initial fitting, for each measure I removed 1–2 outliers with the largest studentized residuals (i.e., > 3.0). After refitting, I examined a plot of residuals versus fitted and a normal probability plot to identify trends in residuals, which were lacking for all models.

To estimate prediction error, which is useful itself but also provides a model-selection criterion, I completed a 10-fold cross-validation using the cv.lm function in the DAAG package in R. The output is the mean square error (MSE) of predicted estimates, and its square root is the root mean square error (RMSE), a prediction analog of the standard error of the estimate in fitted regression equations. Prediction error from cross-validation is asymptotically equivalent to Akaike’s information criterion (AIC), a commonly applied model-selection criterion [58], but low prediction error is most germane for this application.

Do sample-size variables improve these models? To test this, I redid the regressions with three sample-size predictors (sample area, total scarred trees, scarred trees/100 ha) in addition to each of the 21 estimators in the previous models. This time I used best-subset regression in Minitab 17 [51], and the best predictor models were chosen by the lowest Mallow’s *C*_*p*_ statistic, where each included variable also had to be significantly (α = 0.05) related to FR-total scarred trees/plots. I again removed 1–2 outliers based on studentized residuals and examined histograms of residuals and normal probability plots, but found no trends in residuals.

## Results

### Bias, inaccuracy, and regression models to estimate PMFI/FR

Bias was significantly different from 0.0 for all estimators except mean ITFI and inaccuracy was significantly different from 0.0 for all estimators (Table 1). Mean RMEs of -69% to -75% for CFI-all fires, -60% to -69% for CFI-10% scarred, and -38% to -49% for CFI-25% scarred estimators, combined with low standard errors, show that CFI measures all lead to estimates of PMFI/FR that are consistently too short (Table 1). Bias diminished from CFI-all to CFI-25%, but all estimators, except mean ITFI, were still biased. Inaccuracy for CFIs had similar magnitudes, patterns, and trends, with the best still having inaccuracies of 40–50%. ITFI estimators had lower bias and inaccuracy than CFI measures, with bias ranging from -3 to -30% and inaccuracy ranging from 16–33%. Only mean ITFI was unbiased, but still had 30% inaccuracy. FR-recorders also produced significantly biased and inaccurate estimates of FR, averaging 27% too low.

**Table 1:** Bias and inaccuracy in composite fire interval (CFI) and individual-tree fire interval (ITFI) estimates if used to estimate fire rotation-total trees/plots within the 96-case calibration dataset.

Measure	Test of bias					Test of inaccuracy			
	n	Mean RME (%)	s.e. of mean (%)	t	p	Mean RMAE (%)	s.e. of mean (%)	t	p
Mean CFI—all fires	84	-69.35	2.11	-32.79	<0.001	69.42	2.09	33.27	<0.001
Median CFI—all	76	-70.10	2.62	-26.77	<0.001	70.22	2.57	27.27	<0.001
Weibull Scale CFI—all	58	-69.25	2.28	-30.41	<0.001	69.25	2.28	30.41	<0.001
Weibull Mean CFI—all	58	-72.10	2.03	-35.47	<0.001	72.10	2.03	35.47	<0.001
Weibull Median CFI—all	58	-75.46	1.92	-39.33	<0.001	75.46	1.92	39.33	<0.001
Mean CFI—10% scarred	61	-63.29	2.08	-30.46	<0.001	63.39	2.03	31.27	<0.001
Median CFI—10%	62	-68.69	2.14	-32.11	<0.001	68.79	2.09	32.95	<0.001
Weibull Scale CFI—10%	57	-60.09	2.11	-28.48	<0.001	60.09	2.11	28.48	<0.001
Weibull Mean CFI—10%	57	-63.84	1.86	-34.29	<0.001	63.84	1.86	34.29	<0.001
Weibull Median CFI—10%	57	-68.03	1.85	-36.70	<0.001	68.03	1.85	36.70	<0.001
Mean CFI—25% scarred	71	-42.12	2.28	-18.43	<0.001	43.19	1.98	21.82	<0.001
Median CFI—25%	65	-48.88	2.66	-18.35	<0.001	50.77	2.04	24.91	<0.001
Weibull Scale CFI—25%	56	-38.41	2.64	-14.54	<0.001	40.24	2.09	19.30	<0.001
Weibull Mean CFI—25%	56	-44.66	2.35	-19.00	<0.001	45.92	1.86	24.74	<0.001
Weibull Median CFI—25%	56	-49.11	2.24	-21.90	<0.001	49.88	1.91	26.14	<0.001
Mean ITFI	67	-2.71	6.76	- 0.40	0.689	30.10	5.66	5.32	<0.001
Median ITFI	66	-29.71	2.75	-10.79	<0.001	33.43	1.99	16.78	<0.001
Weibull Scale ITFI	56	-8.50	2.53	-3.35	0.001	16.34	1.69	9.65	<0.001
Weibull Mean ITFI	56	-16.64	2.31	-7.22	<0.001	21.19	1.48	14.33	<0.001
Weibull Median ITFI	56	-28.25	2.10	13.43	<0.001	29.68	1.71	17.37	<0.001
FR–recorders	52	-26.79	2.01	-13.31	<0.001	26.79	2.01	13.33	<0.001

Prediction error and fit show that the best regression models to predict PMFI/FR-total scarred trees/plots (Table 2) were from ITFI estimators, particularly Weibull mean ITFI (RMSE = 7.52, *R*^2^_adj_ = 0.972), Weibull scale ITFI (RMSE = 8.04, *R*^2^_adj_ = 0.970), and Weibull median ITFI (RMSE = 9.46, *R*^2^_adj_ = 0.958), although the mean ITFI model was also good (RMSE = 10.30, *R*^2^_adj_ = 0.944). Models based on CFI-25% scarred measures had moderately low prediction errors (RMSE from 11.0–13.7) and high *R*^2^_adj_ values of 0.870–0.929. Models using CFI-10% had higher prediction errors and somewhat lower fit (Table 2). The poorest models were from CFI-all measures (Table 2). Weibull mean models consistently had lowest prediction errors and highest *R*^2^_adj_ compared to models based on mean, median, Weibull scale, or Weibull median (Table 2).

**Table 2:** Linear regression models for estimating PMFI/FR-total scarred trees/plots, based on the 96-case calibration dataset. All slopes (*ß*) were significant (*p* < 0.001) at α = 0.05.

Estimator	*ß* †	Outliers‡	*n*	*R*^*2*^_*adj*_	*RMSE_§_*
Mean CFI—all fires	2.440	25, 89	82	0.721	18.14
Median CFI—all fires	2.450	25, 89	74	0.675	18.52
Weibull Scale CFI—all fires	2.655	25, 93	56	0.755	19.05
Weibull Mean CFI—all fires	2.915	25, 93	56	0.762	18.63
Weibull Median CFI—all fires	3.294	25, 93	56	0.730	20.12
Mean CFI—10% scarred	2.467	25, 89	59	0.837	15.65
Median CFI—10% scarred	2.783	25, 89	60	0.812	16.34
Weibull Scale CFI—10% scarred	2.423	25, 93	55	0.856	16.09
Weibull Mean CFI—10% scarred	2.666	25, 93	55	0.865	15.39
Weibull Median CFI—10% scarred	2.992	25, 93	55	0.826	17.66
Mean CFI—25% scarred	1.715	2, 89	69	0.923	11.00
Median CFI—25% scarred	1.834	26, 89	63	0.870	13.67
Weibull Scale CFI—25% scarred	1.597	2	55	0.925	11.96
Weibull Mean CFI—25% scarred	1.749	2	55	0.929	11.36
Weibull Median CFI—25% scarred	1.867	2	55	0.906	13.00
Mean ITFI	1.121	2, 70	65	0.944	10.30
Median ITFI	1.366	24, 26	64	0.896	12.57
Weibull Scale ITFI	1.108	2	55	0.970	8.04
Weibull Mean ITFI	1.216	2	55	0.972	7.52
Weibull Median ITFI	1.361	2	55	0.958	9.46
PMFI/FR-recorders	1.337	None	52	0.961	10.39

† All models have the form: PMFI/FR-total scarred trees/plots = *ß* * predictor

‡ Numbers represent row numbers in the 96-case calibration dataset (S1 Table)

§ RMSE = root mean square error, the prediction error, in years, from the 10-fold cross validation

Sample-size variables were not significant in most models (Table 3). The few models with significant sample-size variables had *R*^2^_adj_ values generally improved only slightly, averaging higher by only 0.006–0.010 except for the model for mean CFI-all fires, which was 0.086 higher (Tables 2 and 3). Thus, simpler models in Table 2 should suffice for estimating PMFI and FR, except that the sample-size model may be worth using in the case of mean CFI-all fires (Table 3).

**Table 3:** Best linear regression models for estimating PMFI/FR-total scarred trees/plots, including estimators in Table 2 plus measures of sample size, based on the 96-case calibration dataset. Only cases where sample-size variables were significant are shown here, otherwise the best models are in Table 2.

Estimator	Best model	*n*	*R*^*2*^_*adj*_
Mean CFI—all fires	1.817 Mean CFI-all + 0.000896 Sample area (ha) + 0.927 Scarred Trees/100 ha	82	0.807
Mean CFI—10% scarred	2.347 Mean CFI-10% scarred + 0.0447 Scarred Trees	59	0.847
Mean ITFI	1.178 Mean ITFI—0.037 Scarred Trees	65	0.951
Median ITFI	1.260 Median ITFI + 0.360 Scarred Trees/100 ha	64	0.902
PMFI/FR-recorders	1.281 FR from recorders + 0.0702 Scarred Trees	52	0.966

Using prediction error (RMSE) as the criterion, supplemented by fit (*R*^2^_adj_), the best model (Table 2) is based on Weibull mean ITFI, which had the lowest RMSE of 7.52 years and the highest *R*^2^_adj_ of 0.972 (Table 2). The Weibull mean ITFI model was thus used for all PMFI/FR estimation for the 252-site dataset. Given its 7.52 year RMSE, 15–20 year bins are appropriate for reporting estimates, as about 68% of predictions are expected to be within the ± 1 RMSE of 7.52 years. Models other than the Weibull mean ITFI model (Tables 2 and 3) can also be used for deriving estimates from CFI and ITFI estimates, assuming prediction error and fit are acceptable.

### Estimated historical PMFI/FRs across the 342-site dataset

**Fig 3:**
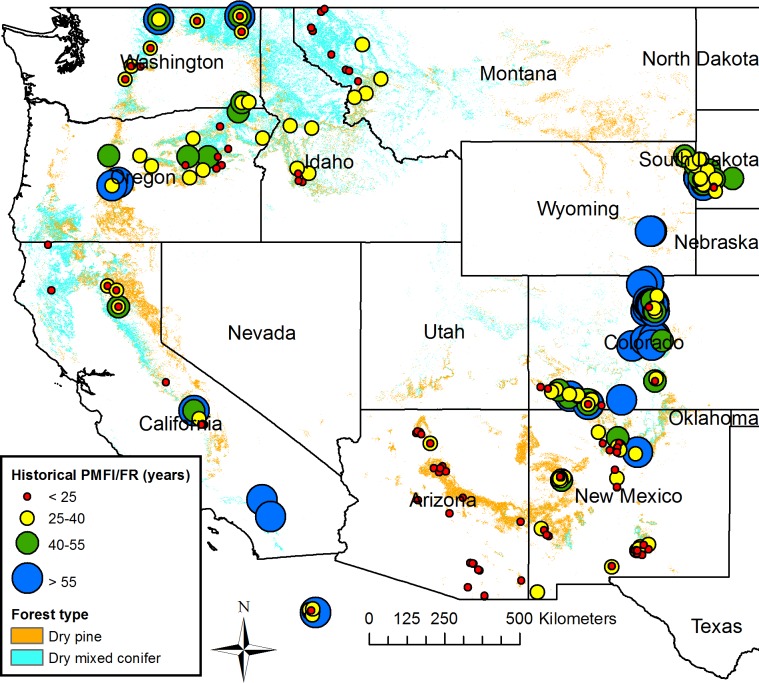
Estimated historical low-severity population mean fire interval/fire rotation (PMFI/FR) for the combined set (n = 342) of calibration cases and prediction sites in dry forests of the western USA.

**Fig 4:**
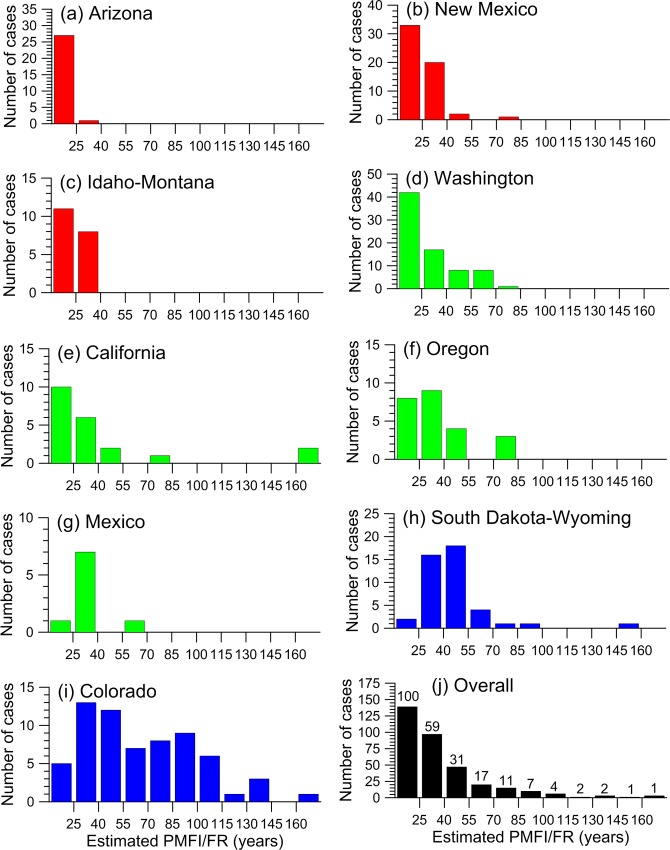
Histograms showing the variability in historical PMFI/FRs (342 sites). These are shown among: (a-i) the eleven western states and (j) overall. In (j) the numbers above the bars indicate the percentage of the distribution that exceeds the lower limit of each bin. For example, 59% of the distribution had historical PMFI/FR > 25 years. Idaho and Montana were combined, as were South Dakota and Wyoming, because of insufficient samples and similarity of histograms within these pairs of adjoining states. Colors indicate similar histograms, with the shortest historical PMFI/FRs predominating in Arizona, New Mexico, and Idaho-Montana, intermediate in Washington, California, Oregon, and Mexico, and the longest in South Dakota-Wyoming and Colorado.

Overall, estimated historical PMFI/FR across the 342 sites had a mean of about 39 years and a median of about 30 years (Table 4). Mean PMFI/FR did not differ significantly between dry pine forests and dry mixed-conifer forests (Table 4; *t* (181) = -0.34, *p* = 0.731). Maps and histograms show that shorter historical PMFI/FRs (< 25 years) were concentrated in Arizona and New Mexico, but also were scattered across parts of all other states, except for few in South Dakota, Wyoming, Colorado, and Mexico (Figs 3 and 4). Historically long PMFI/FR (> 55 years), in contrast, were common only in a band from northern New Mexico to western South Dakota, and were otherwise only scattered in a few locations in California, Oregon and Washington, with no occurrences in Idaho and Montana (Figs 3 and 4). Variability in historical PMFI/FRs was substantial but generally modest within a state, with coefficients of variation (CV) typically between about 30–60%, although California had a high CV and Arizona had a low CV (Table 5). Minima were typically 7–15 years except 20–30 years in South Dakota, Wyoming, and Mexico. Maxima were not very indicative, as a few long PMFI/FR were not uncommon (Fig 4). However, the 3^rd^ quartile of about 93 years in Colorado, 56 years in Wyoming, and 50 years in South Dakota suggests that long historical PMFI/FRs were common in the southern Rocky Mountains and Black Hills (Table 5, Fig 3). At the state level, Colorado stands out in having the greatest variability and total range in historical PMFI/FRs (Fig 4I), and Arizona stands out as having the lowest variability and total range (Fig 4A).

**Table 4:** Overall statistics for historical low-severity PMFI/FR in dry forests and by forest type, based on the merged 342-site dataset. Sample size was 342 overall, 223 in dry pine, 119 in dry mixed conifer.

Statistic	Overall (years)	Dry Pine (years)	Dry Mixed Conifer (years)
Mean	38.62	39.11	37.69
95% confidence interval for mean	35.13–42.10	35.40–42.83	30.42–44.97
Standard deviation	32.75	28.17	40.08
Minimum	7.20	7.20	10.21
1^st^ quartile	19.55	18.80	21.24
Median	29.68	29.95	29.20
95% confidence interval for median	27.01–31.70	26.40–34.63	25.07–31.58
3^rd^ quartile	46.11	50.49	37.62
Maximum	327.16	175.09	327.16

**Table 5:** Statistics for historical low-severity PMFI/FR in dry forests by state, based on the merged 342-site dataset. Sample sizes were 28 in AZ, 21 in CA, 65 in CO, 7 in ID, 12 in MT, 56 in NM, 24 in OR, 40 in SD, 76 in WA, 3 in WY, 9 in MX and 1 in BC.

Statistic	AZ (yrs)	CA (yrs)	CO (yrs)	ID (yrs)	MT (yrs)	NM (yrs)	OR (yrs)	SD (yrs)	WA (yrs)	WY (yrs)	MX (yrs)	BC (yrs)
Mean	15.48	54.21	65.70	25.96	21.81	24.59	36.41	46.19	30.60	47.18	35.04	40.49
s.d.	4.26	83.01	35.32	8.28	6.77	11.24	19.09	22.23	16.09	14.92	13.06	-
CV	27.52	153.13	53.76	31.90	31.04	45.71	52.43	48.13	52.58	31.62	37.27	-
Minimum	7.20	8.56	15.20	16.88	13.25	10.21	15.30	21.18	11.00	29.95	23.15	40.49
1^st^ quartile	12.51	18.68	35.05	17.00	14.88	16.25	24.12	35.20	19.73	29.95	28.62	-
Median	15.22	27.20	60.45	27.07	22.47	22.28	29.66	41.84	23.89	55.79	32.28	40.49
3^rd^ quartile	17.98	40.77	92.67	32.95	26.95	30.62	42.33	49.97	38.30	55.79	35.88	-
Maximum	25.70	327.16	175.09	37.37	32.83	74.70	83.25	158.70	81.93	55.79	68.08	40.49

Another pattern is that in the most mountainous areas with the steepest environmental gradients and topographic diversity, the full range (all four classes) in historical PMFI/FRs often was found in a small area (Fig 3). This high diversity occurred in northeastern Washington, the central Sierra Nevada, northern New Mexico, southwestern Colorado, north-central Colorado, and in western South Dakota, but not in Arizona, Idaho, Montana, or Oregon (Fig 3). However, even in these areas, with the exception of Arizona, some diversity in historical PMFI/FR was found over relatively short distances (Fig 3), suggesting the importance of local factors in addition to the large trends evident across the western USA.

**Fig 5:**
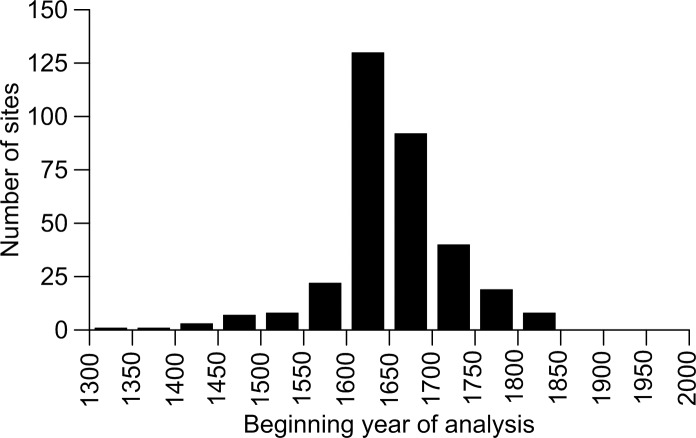
Beginning year of analysis for the 331 sites with available data in the 342-site merged dataset.

Most studies of low-severity fire in dry western forests were conducted in forest stands that were mostly old forests in the pre-EuroAmerican era (Fig 5). Stands with beginning analysis years before A.D. 1750 were likely generally ≥ 150 years old, and stands with beginning analysis years before A.D. 1700 were likely generally ≥ 200 years old, in A.D. 1900, thus meeting the age criterion for old-growth forests (Fig 5). A history of predominantly low-severity fire in the century before the late-1800s was found across about 34%, on average (ranging from 2.5–62.4%), of eleven dry-forest landscapes across the western USA (Table 6). Thus, estimated historical PMFI/FRs apply primarily to old forests, which were likely concentrated historically in the 34% of overall dry-forest landscapes with a history of predominantly low-severity fire.

**Table 6:** Area and percentage of 11 dry-forest landscapes in the western USA that meet the low-severity model, based on GLO surveys and early aerial photographs.

Source/Author(s)	Study area		Fits low-severity model	
	Location	Area (ha)	%	Area (ha)
*General Land Office Surveys*				
Williams and Baker [53]	Mogollon Plateau, AZ	405,214	62.4	252,854
	Black Mesa, AZ	151,080	12.0	18,130
	Front Range, CO	65,525	2.5	1,638
	Blue Mountains, OR	304,709	40.3	122,798
Baker [59]	North-E Cascades, OR	146,555	32.5	47,630
	Central-E Cascades, OR	147,502	10.4	15,340
	South-E Cascades, OR	104,160	29.4	30,623
Williams and Baker [60]	Coconino Plateau, AZ	41,214	58.8	24,234
Baker [61]	N. Sierra, CA	115,766	12.6	14,587
	S. Sierra, CA	187,085	26.4	49,390
*Early Aerial Photographs*				
Hessburg et al. 2007	E. WA & E. OR	112,115	21.6	24,200
*Symopsis*				
Total		1,780,925		601,424
Mean percentage			33.8	

## Discussion

### Limitations of CFIs and ITFIs if used to estimate PMFI/FR

Researchers in the past commonly sampled fire scars and trees to generally increase the length of the fire-history record, minimize physical damage to trees, and maximize efficiency [55]. Unfortunately, these methods also produced CFI and ITFI estimates that are biased and inaccurate if used to estimate the PMFI/FR (Table 1), as also found from modern calibration [11], and this is now accepted to be an inappropriate use. However, it is possible to estimate PMFI/FR accurately from past CFI and ITFI estimates using linear regression (Table 2).

Further discussion of the limitations of past measures as estimators of the PMFI/FR is thus generally moot, but for those interested, I include further analysis in S1 Text and a summary here. The main factors unique to underestimation of PMFI/FR by CFI measures likely include: (1) overcompensation—sampling and compositing across too large an area, (2) loss of long real fire intervals to the compositing process, and (3) restriction rules that do not omit enough small fires. ITFI measures do not use compositing and have lower bias and inaccuracy, but still are biased and inaccurate (Table 1). Both CFI and ITFI measures must be missing longer intervals from a sampling bias, because their estimates are low relative to PMFI/FR. Major factors likely are targeting trees and sampling areas with the most scars, excluding trees with no or one scar, and censoring intervals at the beginning and end of a tree’s record (S1 Text). Targeting can also reduce estimated PMFI/FR itself (S1 Text), and needs to be avoided in new landscape methods.

### Inference space for the PMFI/FR estimates

Studies of new probabilistic landscape methods for reconstructing PMFI/FR encourage “… clearly defining the inference space, not extrapolating to unrepresentative areas …” ([55]:1030), and this is also important for estimates of PMFI/FR from regression. The dataset of 342 sites spans dry forests in the western USA (Fig 1). The set of published studies corresponding to this dataset (S2 Table) includes many of the studies of low-severity fire in dry forests in the western USA, but other studies exist. This dataset and these other studies likely are not a probabilistic sample of historical dry forests, however, as many studies targeted old forests or forests with concentrations of fire scars (S1 Text) and occurred in forests that likely were old in the pre-EuroAmerican era (Fig 5). Old trees were historically dominant in some dry-forest landscapes and old trees were not uncommon in many forests, but young to middle-aged forests historically dominated most dry-forest landscapes [24, 62, 63]. Based on the GLO reconstructions and early aerial photographs (Table 6), the PMFI/FR estimates here apply most clearly to no more than about 34% of dry-forest landscapes, particularly in old forests. That leaves about 66% of dry-forest landscapes without PMFI/FR estimates. It is possible that some estimates do apply to parts of these other forests, possibly representing the low-severity parts of mixed-severity fire regimes on sites that had not recently burned at high severity. However, it is impossible to determine this from data in FHX files or for the 74% of studies that did not reconstruct fire severity (S1 Text).

Several studies, that targeted old forests to obtain long fire records, indicated that younger forests had few fire scars and, because these studies were focused on long and complete records of fire years, they avoided sampling younger stands. In El Malpais, New Mexico: “The most abundant, best preserved fire-scarred samples were found at sites on the northwestern and western peripheries of the malpais … We found no fire-scarred samples on the kipukas in the northern and eastern portions of the malpais, and found few samples in the southern portions. These areas contained ponderosa forests that appeared younger than elsewhere, perhaps due to more recent, intense stand-replacing fires …” ([64]:136). Sampling was concentrated in areas with abundant fire-scars, but later this targeting was forgotten, and these areas were portrayed as representing the whole El Malpais landscape: “These increased fuel loadings in malpais forests have essentially changed the trajectory of fire behavior to one that now favors the occurrence of high-intensity, stand-replacing fires in contrast to the low-intensity, stand-maintenance fires that occurred prior to Euro-American settlement …” ([64]:234). Similarly, no fire scars were found in 5 of 12 transect locations in mixed-conifer forests in northern New Mexico [65]. The study sampled scars on relatively flat ridges nearby, where scars were abundant, and composite fire intervals from these sites were assumed to apply to the whole mixed-conifer forest [65]. This was also the pattern in northern Colorado: “Most of the 67 fire-scarred trees that were sampled were found on ridges or in open areas (Fig 1). It was uncommon to find scarred trees in dense stands” ([66]:138).

These observations suggest low-severity fire was likely less frequent or even rare in younger and denser historical dry forests, that likely were common in the 66% of dry forests lacking a history of exclusive low-severity fire (Table 6). However, specific studies of rates of low-severity fire are lacking for stands ≤ 150–200 years old in the pre-EuroAmerican era (Fig 5), that are the predominant forests today. Because they are not in the inference space for past fire-history studies in dry forests, it is not valid to infer that today’s young to middle-aged forests would have been subject to low-severity fires at the historical mean rates in the 342-site dataset or in other comparable published fire-histories for dry forests.

### Historical dry forests not predominantly frequent-fire forests

Dry pine and dry mixed-conifer forests have been described as frequent-fire forests, an attribute still supported for only about 14% of overall dry-forest area, with multidecadal low-severity fire likely historically over about 86% of overall dry-forest area in the western USA. Only about 41% of the old, dry forests, which were likely concentrated in about 34% of western USA dry forests (41% of 34% = 14% of overall dry forest), had frequent fire, with a historical PMFI/FR < 25 years (Fig 4J). Old forests with frequent fire were historically concentrated in Arizona and found at scattered sites across the West (Fig 3), particularly in New Mexico, Washington, Idaho, Montana, and California (Fig 4A–4E). In contrast, about 59% of cases in old forests and thus about 20% of dry forests in the western USA (59% of 34% = 20% of overall dry forest) had a historical mean PMFI/FR ≥ 25 years (Fig 4J). Low-severity fire was likely even less frequent in the remaining overall 66% of dry-forest landscapes lacking a history of exclusive low-severity fire (Table 6). Altogether roughly 14% of dry forests in the western USA historically had frequent (PMFI/FR < 25 years) low-severity fire and 86% of dry forests in the western USA historically instead had multidecadal low-severity fire.

Even in the 34% of dry-forest landscapes with an exclusive history of low-severity fire, the overall mean PMFI/FR was 39 years, half the cases had PMFI/FR > 30 years, and a quarter of cases had PMFI/FR > 46 years (Table 4). These old forests are better described overall as having diverse rates of low-severity fire, spanning the range from frequent to multidecadal. This diversity in rates varied on two scales, first across large regions from predominantly multidecadal (median > 40 years), in Colorado, South Dakota, and Wyoming, to predominantly frequent in Arizona, New Mexico, and Idaho-Montana, with other states having broader mixtures, ranging from frequent to multidecadal (Figs 3 and 4). Second, individual smaller areas often contained a diversity of rates over short distances, particularly in mountain ranges, often spanning or nearly spanning a broad range from frequent to multidecadal (Fig 3).

Estimated historical PMFI/FR mean rates are relevant, because many ecological processes and structures change across a narrow range in rates. In the roughly 86% forests with PMFI/FR ≥ 25 years, fuels that required about 7–25 years to build back up after a low-severity fire [12–14]. would, on average, have been fully recovered for an extended period before the next fire. Shrubs would likely have been able to fully recover and dominate for substantial periods. Small trees that rely on seed (e.g., ponderosa pine) would also have been able to regenerate and become common in forest understories, as documented in several historical dry forests [24]. The role of the forest floor in replenishing soil nutrients and organic matter, enhancing absorption of water, and fostering microbial communities [15] would not have been limited by too-frequent fires. Greater opportunities for trees to regenerate and less mortality from low-severity fire also help to explain dense areas of dry forests that occurred historically across substantial parts of many dry-forest landscapes (e.g. [53, 59]). Natural fuels, less limited by low-severity fire, would have favored higher-severity fires via ladder fuels. Adverse effects on habitat for wildlife that use snags or coarse down wood [15] would be less because of less low-severity fire, and fires of higher intensity would likely increase snags and coarse dead wood.

In contrast, in the roughly 14% of historical dry forests with historical PMFI/FR < 25 years, levels of fuels, including shrubs and small trees, would have been more consistently kept low (Fig 3). Frequent low-severity fires would likely have fostered a diversity of grasses and forbs, but would have limited shrubs and small trees. In these settings, lower-density forests would have been favored and higher-severity fires would have been discouraged, at least by fuel conditions [19, 53]. Potential adverse ecosystem and wildlife effects of frequent low-severity fire [15] would remain a natural historical characteristic of these primarily southwestern frequent-fire forests (Fig 3). However, high local and regional diversity in rates (Fig 3) meant that a diversity of processes, rates, and structures occurred across even the old-forest part of many dry-forest landscapes, within both small areas and across the western USA.

### Limitations and error in calibration and prediction PMFI/FRs

The calibration cases (S1 Table) are from larger land areas and include estimates of PMFI/FR that are directly usable as a guide for restoration and management in old, dry forests. The appropriate estimate in S1 Table is FR-YrsTot, which was directly estimated in the study in many cases. Where a direct estimate was not made, I estimated PMFI/FR-YrsTot from PMFI/FR-YrsRec using the equation in Table 2 and Fig 2B.

The 252 prediction cases (part of S2 Table) are from single-plot samples in smaller plot areas, and likely have more error. The estimated prediction error for PMFI/FR in a small plot was a 7.52 year RMSE, which suggests bins about 15-years wide, as in Fig 3, would likely contain about 68% of observations. Bins about 30-years wide would contain about 95% of observations. Smaller plots used at the 252 sites also may not individually provide an adequate sample of a forest area. In an accuracy study, estimates from small plots required averaging across 5–6 plots representing 600–1000 ha to achieve mean relative errors < 30% in estimating PMFI/FR [11]. The estimated PMFI/FRs from the available set of small plots cannot be pooled to decrease this error, as they are not necessarily samples from one population. The problem for small plots is inherent stochastic variability in realized fire intervals, even from a fixed fire regime in a particular land area [67], and errors in the sample and estimators. Thus, the PMFI/FR estimates are a significant improvement over using CFI and ITFI, but greater accuracy can be expected from larger studies in the calibration dataset and also from future landscape-scale reconstructions.

Most of the 342 estimates are likely low estimates for two reasons. Targeting multi-scarred trees reduces CFI and ITFI estimates, but also reduces estimated PMFI/FR by not sampling trees with one scar or no scar that can indicate areas that did not burn in a particular fire (S1 Text). Thus, the area burned by each fire may be inflated and the PMFI/FR too short. Because 94% of 250 cases with evidence did target multi-scarred trees (Table A in S1 Text), this affects almost all estimates of PMFI/FR. Targeted sampling of individual trees led to PMFI/FR estimates reduced to about 86–95% of estimates from equal-sized probabilistic samples [55]. This would mean that PMFI/FR estimates here need to be multiplied by 1.05–1.18. Also, both calibration and prediction PMFI/FR estimates are low estimates in many cases because PMFI/FR could not be estimated separately for low-severity fires in the 74% of cases where fire-severity was not studied. Even where fire severity was studied, the study did not report separate rates, instead only rates for fire severities combined (S1 Text). Because estimates are for old forests with a history of low-severity fire, the higher-severity component was likely not large, but could affect longer estimates (Table B in S1 Text). Combining these two factors likely would increase estimated PMFI/FR, but more research is needed to narrow and validate the needed corrections before they are applied. In contrast, FR estimates in the calibration are from all trees, not recorders, and regression equations applied to the predicted dataset are from all trees. Estimates from recorders would be lower, but I explained earlier why the truth is likely closer to FR-all trees. Further research is warranted, and could possibly resolve all remaining uncertainties, leading to improved equations and estimates.

### PMFI/FRs as a guide to restore and manage low-severity fires

In spite of these limitations, these new PMFI/FR estimates are the best available and usable estimates of historical mean rates of low-severity fire to use as a guide in restoring and managing low-severity fire in dry forests of the western USA. Past CFI and ITFI estimates were not intended to estimate PMFI/FR and would be misapplied, with adverse impacts on biological diversity and ecosystem functioning, if used directly for this purpose, as is shown by their biases, inaccuracies, and needed adjustments using regressions (Tables 1, 2).

Estimated historical PMFI/FRs specify how long, on average, it took to burn across a land area (the FR), and how long the intervals were, on average, between fires at points in the land area (the PMFI). They can be estimated at multiple scales, from small plots to large land areas, although with greater accuracy over larger land areas. Congruent estimates of modern and historical low-severity PMFI/FR can be made, and directly compared. Modern estimates can be made using digital fire maps (e.g., Monitoring Trends in Burn Severity at: http://www.mtbs.gov) or other sources. All that is needed is to add up the areas of fires that burned in a particular landscape of interest at low severity over a particular period, calculate the area of the landscape, and use Eq 2. Temporal and spatial variability in PMFI/FR can be estimated as well, using subareas or sub-periods (e.g. [23]). Comparison of modern and historical rates of low-severity fire facilitates monitoring the effectiveness of restoration and management programs, and analysis of trends in rates of modern relative to historical fires [3].

**Fig 6:**
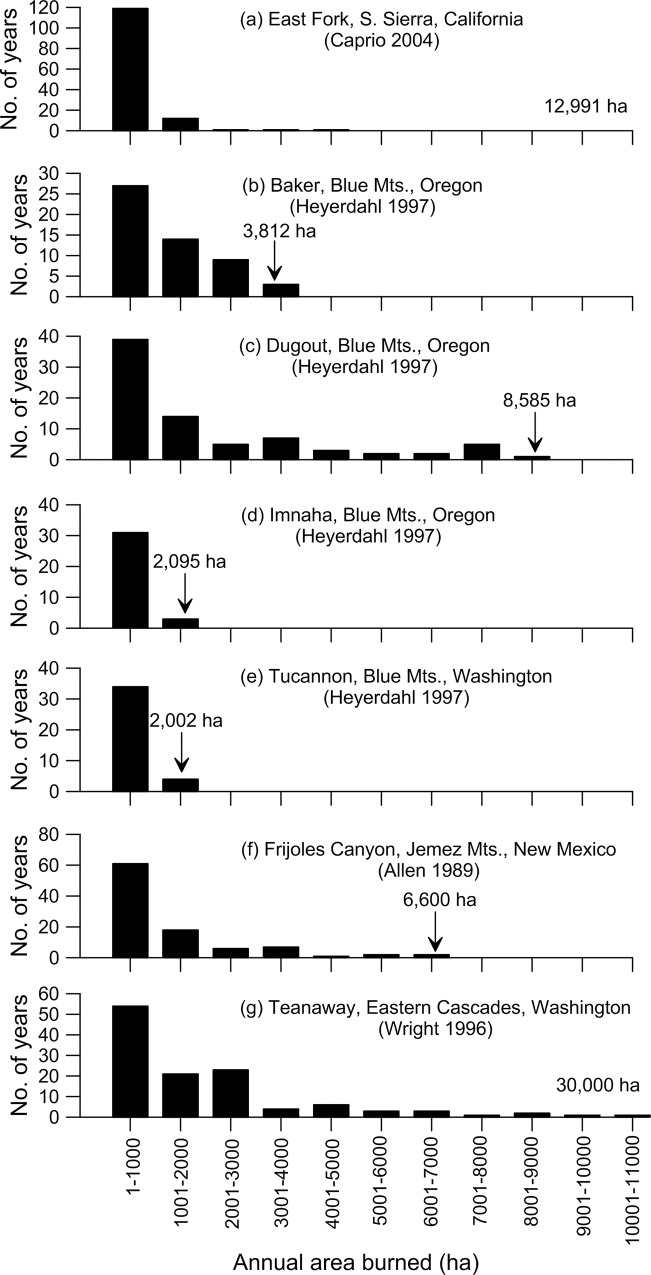
Size distribution for historical annual area burned in seven large study areas in dry forests of the western USA. Study area size is given above arrows or at the right of the x-axis.

Fire-size distributions are also important, but those from small plots have inherently limited value. At this point, distributions of annual area burned, which approximate fire sizes, can be shown for some larger study areas in dry forests (Fig 6). I compiled data for these histograms from graphs or tables in the sources. Note that this is area burned at all severities, not just low severity, and is not restricted to old-forest parts of landscapes. Several graphs show that the most fire years were in the smallest size class, with decreasing abundance in larger size classes. Historical fire sizes could reach at times into the 5,000–11,000 ha size classes, at least in three study areas (Fig 6C, 6F and 6G). In many study areas, the maximum area burned reached the size of the study area (Fig 6B, 6C, 6D, 6E and 6F), suggesting fires could have been larger.

In an Arizona dry-forest landscape, 5.1% of total fires, that were the largest fires, contributed 97% of total burned area [1]. This pattern, common in forests [17], also suggests that in dry forests most of the burned area is from infrequent large fires, with frequent small fires not adding much to total burned area. This pattern of variable fire sizes and infrequent large fires is important to mimic, as it fosters diverse times since fire, at any instant, across a landscape, which allows species with different responses to fire to all remain viable across landscapes [16].

Low-severity fires can kill up to about 20% of basal area [27–28], and it is usually expected that this mortality is from torching or passive crown fires that kill individual trees or small groups of trees. However, little is known about the size and distribution of patches of mortality in low-severity fire regimes. Only about 23% of reconstructions of low-severity fires analyzed fire severity and even these provided little information about this topic (S1 Text), as it is difficult to reconstruct the size of mortality patches. Early historical observations provide some evidence. For example, generally low-severity fires in Sierran mixed conifer forests were observed to also have about 15% high-severity fire in small patches [20]. Small high-severity patches from low-severity fires in these forests were described [68] as mostly < 2–4 ha ([61]:Table A1 Q18-Q25). More research is needed on early historical observations of canopy mortality from low-severity fires, but some is historically congruent and expected from low-severity fires in modern forests.

Unburned areas within the perimeters of fires are also important for biological diversity and natural recovery, as these areas serve as refugia for less fire-tolerant plants or those that regenerate by seed, facilitating survival in these areas and natural recovery within the burned area [69]. This study found 35% average unburned area inside 154 modern fire perimeters in Yosemite National Park, California, which included substantial dry-forest area. Unfortunately, little is known about the extent of unburned area in historical low-severity fires in dry forests. It is known that prescribed burning that fully blackens burn units can reduce spatial heterogeneity in fire that promotes coexistence of multiple species [16]. Also, as reviewed in the introduction, unburned areas historically were locations where tree regeneration to replace tree mortality could survive. Thus, including unburned area within burn units, rather than blackening the whole unit, is ecologically important to restore and maintain tree populations and biological diversity.

The extent of needed burning to restore and manage old dry forests and the rest of dry forests is lower than previously thought. Earlier estimates were largely based on the assumption that reported mean CFI estimates represent PMFI/FR, which they do not (Tables 1, 2) and apply to all dry forests, which they also do not. Estimated historical rates of low-severity fire in dry forests in the U.S. Landfire program, for example, typically incorrectly use reported mean CFI estimates as though they represent PMFI/FR, although some actual PMFI/FR estimates are also used. These are applied to all dry forests, not just old forests. Both misapplications likely have adverse effects on biological diversity and ecosystem functioning. Prescribed burning in U.S. national forests, national parks, and on other public lands, where Landfire or other estimates from mean CFIs have been used as a guide, is likely too much by 1.6–3.3 times, depending on the CFI measure used (See *ß* inTable 2) in the roughly 14% of dry-forest area that was historically old forest with frequent fire (PMFI/FR < 25 years). Mean rates are likely too high by > 1.6–3.3 times in the 86% of the area of dry-forest landscapes that historically had multidecadal low-severity fire.

A need for less low-severity fire in restoration and management of dry forests is good news, because costs of prescribed burning and other restoration treatments are high, effects on invasive species, ecosystem processes, and biological diversity are a concern [15, 70], and the feasibility of restoring and managing low-severity fire is higher with longer rates. Longer rates also mean that completed treatments may have already been sufficient in many old-forest areas, and further management of low-severity fire can be redirected to using managed fire for resource benefit [71]. Where initial treatment is incomplete, one prescribed fire should suffice before a managed-fire program can begin. At that point, managers can monitor low-severity fire using historical mean PMFI/FR rates, fire-size distributions, and other attributes (e.g., unburned area) as a guide.

In locations where managed fire for resource benefit is infeasible, and an ongoing prescribed-burning program must be used, burning at rates longer than the mean PMFI/FRs reported here and using a diversity of rates and patterns of prescribed fires would be congruent with the findings. First, substantially lower rates (longer PMFI/FR) are warranted, if forests are not old forests, because estimated rates here apply mostly to old forests and the prevailing younger forests today likely burned historically at longer PMFI/FR. Second, the rates reported here are likely somewhat too short, as explained in “Limitations and errors…” Finally, lower rates would likely reduce the spread of invasive species and adverse effects on ecosystem processes and biological diversity. Also, historical rates varied substantially within small areas, particularly where there was topographic diversity, but also because of natural variability over time. It makes sense to similarly vary prescribed burning rates within local areas, leaving some areas unburned for longer periods. An approximation of the percentage of western USA old-forest parts of landscapes that experienced longer historical rates of fire is in Fig 4J. More local data can be derived from S2 Table, which lists PMFI/FR by state.

Data presented here can generally be used, with other evidence and tools, to create more comprehensive and spatially informative local understanding about mean historical PMFI/FR to guide local restoration and management of low-severity fire in old-forest parts of landscapes. Data in S2 Table have latitude and longitude and other ancillary information, and can be downloaded (S2 Dataset) and used directly or be read into a GIS program, where topography, land ownership and other information can be added for context. As new data are added to the IMPD, an FHX file for each new site can be downloaded and read into FHAES. Weibull mean ITFI can be calculated, which can then be used (Table 2) to estimate historical PMFI/FR, if not already provided in the study. Geographical coordinates, usually in the FHX file, allow new data to be added to the database (S2 Dataset) for use in GIS. Estimates of historical mean CFI and ITFI are available in the published scientific literature for other sites, not in the IMPD, which can also be used to estimate historical PMFI/FR using the equations in Tables 2 and 3, then added to the dataset (S2 Dataset) and input into GIS for local analysis. Of course, these estimates usually apply to only old-forest parts of historical landscapes.

Dry-forest landscapes until recently were thought to have historically been primarily old-growth forests, with a history of frequent low-severity fire, across their extent (e.g. [72]), but this has been refuted by GLO reconstructions and early aerial photographs (Table 6), paleoecological evidence [24], and early forest-reserve reports and other evidence [63, 73]. Even in Arizona, which had abundant old forests with frequent fire (Fig 3), denser forests and high-severity fire were extensive at certain times and in certain places, as on Black Mesa and parts of the Mogollon Plateau [60, 73]. It is sensible to restore low-severity fire to its former dominance in the parts of dry-forest landscapes with a history of primarily low-severity fire, historically averaging about 34% of western dry-forest landscapes (Table 6). Estimated mean PMFI/FRs here provide a guide for restoration and management of low-severity fire in extant old-forest parts of landscapes. For most dry-forests today, which are not old, using frequent fire (PMFI/FR < 25 years) in restoration is not supported, and fuels do not need to be substantially reduced, because historical PMFI/FRs naturally allowed historical shrubs and small trees to fully recover after fires. Restoration of low-severity fire is still needed. The most appropriate approach, given likely long but uncertain mean rates of historical low-severity fire, is for most dry forests today to receive at most one prescribed fire, followed by managed fire for resource benefit, with the goal of mimicking mean historical PMFI/FRs and variability in fire (fire-size distributions, unburned area) as forests reach old age.

## Supporting information

S1 Dataset (XLS)

S2 Dataset (XLS)

S1 Dataset metadata (PDF)

S2 Dataset metadata (PDF)

S1 Table Authors, locations, and values for CFI and ITFI estimators and PMFI/FR in the 96-case calibration dataset. (PDF)

S2 Table Authors, sites, the Weibull mean ITFI estimate, and the calibrated or predicted PMFI/FR for the merged 342-site dataset. (PDF)

S1 Text Why CFIs and ITFIs underestimate PMFI/FR. (PDF)
