# Supplementary material for: Restoring and managing low-severity fire in dry-forest landscapes of the western USA
Source: PLoS One. 2017 Feb 15;12(2):e0172288. doi: 10.1371/journal.pone.0172288 (PMC5310858; doi:10.1371/journal.pone.0172288)
Supplement: S1 Dataset metadata — (PDF) [file pone.0172288.s003.pdf]

Metadata–Calibrated Dataset–96 cases

January 30, 2017

The FHX file is the computer file, containing fire-history data, provided by authors to the International Multiproxy Paleofire Database (IMPD), which is located here: https://www.ncdc.noaa.gov/data-access/paleoclimatology-data/datasets/fire-history

The Fire History Analysis and Exploration System (FHAES) is software that reads the FHX file and allow computation of fire-history measures. It can be downloaded here: https://www.frames.gov/partner-sites/fhaes/download-fhaes

THE FIRST THREE COLUMNS DESCRIBE THE SAMPLE
C1 = **Author**, see the bibliographies in the paper
C2 = **Site**, as given in the FHX file from the IMPD or in the publication if there was no FHX file
C3 = **IMPDdataset**, the name assigned to the dataset in the IMPD

THE NEXT SEVEN COLUMNS ARE ABOUT THE SAMPLE SIZE
C4 = **SampleArea(ha)**, as given in the FHX file or, if an FHX file not available, then as given in the publication. In many cases, neither source provided this information.
C5 = **ScarredTrees**, as given in the FHX file or, if an FHX file not available, then as given in the publication. In many cases, neither source provided this information.
C6 = **ScarredTrees/100ha** = C5/(C4/100))
C7 = **TotalScars**, the total number of fire scars that were dated across all the wedges/cores and all the years on each wedge/core, based on the publication. This information was often not provided in the publication.
C8 = **MeanTotalScarredTrees**, calculated from the FHX file as the mean for the Total Samples column in the Summary Table available in FHAES after editing it to limit it to the Time Period (years) of the analysis. Missing data typically occur because the FHX file was a composite file, which cannot be used to produce a Summary Table.
C9 = **TotalEvents**, calculated from the FHX file as the sum of the Events column in the Summary Table available in FHAES after editing it to limit it to the Time Period (years) of the analysis. Missing data typically occur because the FHX file was a composite file, which cannot be used to produce a Summary Table.
C10 = **MeanScars/ScarredTree**, calculated as C9/C8 if an FHX file and a Summary Table in FHAES were available, otherwise calculated as C7/C5 from the publication, if the necessary information was reported. Missing data may occur because the FHX file was a composite file, which cannot be used to produce a Summary Table.

THE NEXT THREE COLUMNS DEFINE THE SAMPLE PERIOD
C11 = **FIAnalysisYears**, which extend from the first fire year in or after A.D. 1600 to the last fire year before or in A.D. 1900 in the case of the 252-site prediction dataset and over any period, from a first fire to a last fire, in the case of the 96-site calibration dataset.
C12 = **FIAuthorsYears**, which are the years analyzed by the authors, as reported in the publication. This information was not always provided.
C13 = **FIBegins**, which is the first year of the author(s)' analysis, as recorded in C12, or the first year of the analysis, as recorded in C11 if the author did not present the analysis period. The

purpose of this field is to allow calculation of the approximate age of the forest.

THE NEXT 15 COLUMNS CONTAIN THE ESTIMATORS
C14 = **CompMeanFIAll**, Composite mean fire interval - all trees
C15 = **CompMedianFIAll**, Composite median fire interval - all trees
C16 = **CompWeibScaleFIAll**, Composite Weibull scale fire interval - all trees
C17 = **CompWeibMeanFIAll**, Composite Weibull mean fire interval - all trees
C18 = **CompWeibMedianFIAll**, Composite Weibull median fire interval - all trees
C19 = **CompMeanFI10%**, Composite mean fire interval - 10% scarred
C20 = **CompMedianFI10%**, Composite median fire interval - 10% scarred
C21 = **CompWeibScaleFI10%**, Composite Weibull scale fire interval - 10% scarred
C22 = **CompWeibMeanFI10%**, Composite Weibull mean fire interval - 10% scarred
C23 = **CompWeibMedianFI10%**, Composite Weibull median fire interval - 10% scarred
C24 = **CompMeanFI25%**, Composite mean fire interval - 25% scarred
C25 = **CompMedianFI25%**, Composite median fire interval - 25% scarred
C26 = **CompWeibScaleFI25%**, Composite Weibull scale fire interval - 25% scarred
C27 = **CompWeibMeanFI25%**, Composite Weibull mean fire interval - 25% scarred
C28 = **CompWeibMedianFI25%**, Composite Weibull median fire interval - 25% scarred
C29 = **IndivTreeMeanFI**, Individual tree mean fire interval
C30 = **IndivTreeMedianFI**, Individual tree median fire interval
C31 = **IndivTreeWeibsScaleFI**, Individual tree Weibull scale fire interval
C32 = **IndivTreeWeibMeanFI**, Individual tree Weibull mean fire interval
C33 = **IndivTreeWeibMedianFI**, Individual tree Weibull median fire interval

THE NEXT COLUMN EXPLAINS THE SOURCE OF THE ESTIMATOR VALUES
C34 = **Fisource**, the source of the estimator values, which can be either: (1) a page number in the publication cited in column C1 and given in the bibliographies in the paper, (2) FHAES, which means that the values were obtained by using FHAES to calculate the values from the FHX file listed in column C3.

THE NEXT 8 COLUMNS CONTAIN THE ESTIMATED FIRE ROTATIONS AND THEIR SOURCES AND METHODS OF CALCULATION
C35 = **FRTotalTrees**, which is the estimated fire rotation calculated using all trees or all plots
C36 = **FRRecorders**, which is the estimated fire rotation calculated using only recorder trees
C37 = **FRMethodCode**, which can be: (1) Author estimate from fire-year maps, fire atlases, or digital spatial fire records, (2) My estimate from area burned in an author table or graph, (3) Author estimate from the ratio method, (4) My estimate from the ratio method and author's FHX file using FHAES, or (5) My estimate from the ratio method using author's data in a table or interpolated from a graph.
C38 = **FRMethod**, which can be: Area burned-graph, Area burned-table, Fire-year map-sites/trees, Fire atlas, Polygon fire records, Ratio-sites-Author, Ratio-trees-Author, Ratio-trees-FHAES
C39 = **AreaBurnedCalculation**, which has notes about how area burned was calculated, when this information was available.
C40 = **FRsource**, which gives the page number where the fire rotation estimates were recorded in the publication or gives FHAES, which indicates that the fire rotation estimates were

calculated in FHAES and Minitab from the FHX file.
C41 = **FRAnalysisYears**, which gives the time period using for the fire rotation calculations
C42 = **FRAuthorsYears**, which gives the time period for the author's analysis of fire history

THE NEXT COLUMN DEFINES THE VEGETATION TYPE
C43 = **VegType**, which is either DryPine, for *Pinus ponderosa* and related pines of dry western forests, or DryMC for dry mixed-conifer forests, which are forests with ponderosa pine or other dry pines dominant but also with other trees (e.g., white fir, Douglas-fir, quaking aspen) present.

THE NEXT 8 COLUMNS ARE ABOUT TARGETTED SAMPLING
C44 = **Targeting**, which lists the page(s) in the publication where targeted sampling, if used, was explained.
C45 = **TargetBestInfoLongestRecord**, which is "Yes" if this form of targeting, which seeks to obtain the longest record of historical fires, was used, "No" if the author explained that this was not used, and is "No explanation" if this form of targeting was not mentioned or specifically explained.
C46 = **TargetMultiScarred**, which is "Yes" if this form of targeting, which favors trees with multiple fire scars, was used, "No" if the author explained that this was not used, and "No explanation" if this form of targeting was not mentioned or specifically explained.
C47 = **TargetClusters**, which is "Yes" if this form of targeting, which favors sampling fire scars in clusters, was used, "No" if the author explained that this was not used, and "No explanation" if this form of targeting was not mentioned or specifically explained.
C48 = **TargetDeadWood**, which may be either "Many" or "Some" if this form of targeting, which favors fire scars on standing dead or down wood, was used, "No" if the author explained that this was not used, and "No explanation" if this form of targeting was not mentioned or specifically explained.
C49 = **TargetSpecies**, whether tree species were preferentially sampled, which may be "Ponderosa pine," "No" if the author explained this form of targeting was not used, and "No explanation" if this form of targeting was not mentioned or specifically explained.
C50 = **TargetPlots**, which may be "Many scarred trees" or "Old forests" or "Ponderosa pine" if plots were targeted in locations with these attributes, "No explanation" if targeting was not used or was not explained, "No plots" if plot-sampling was not used, "Objective-grid" if a non-targeted method based on an objective grid was used, "Objective-random" if a non-targeted method based on random sampling was used, "Proximity/access" if this criterion was used, which is ambiguous regarding objectivity, and "Range of settings/gradient" if the plots were spread across settings or a gradient (e.g., elevation) but it was unclear whether the actual plot placement was objective.
C51 = **TargetStudyAreas**, which may be many explanations

THE NEXT 3 COLUMNS ARE ABOUT WHETHER FIRE SEVERITY WAS STUDIED
C52 = **FireSeverity**, which is "No" if not studied, "Yes" if studied, and "No explanation" if the publication does not explain whether fire severity was studied.
C53 = **FireSeveritySource**, which is "Age structure-plots" if fire severity was determined at the plot scale, "Age structure-plots/aerial photos" if both plots and aerial photos were used, "Age

structure-pools only" if fire severity was determined from a pool of plot data, which is not reliable as it masks locally severe fires, "Inference" if fire severity was inferred, rather than specifically studied, "Inference/dating" if some dating accompanied the inference, "Not studied" if not studied, and "No explanation" if it was not possible to determine whether the author studied fire severity.

C54 = **FireSeverityComments**, which explain some aspects of the fire-severity determinations

THE NEXT 9 COLUMNS ARE ABOUT THE LOCATION OF THE SAMPLE

C55 = **State**, there are these: AZ, CA, CO, ID, MT, MX, NM, OR, SD, WA, WY, plus MX, BC

C56 = **StateCode**, there are these: 1 = AZ, 2 = CA, 3 = CO, 4 = ID, 5 = MT, 6 = MX (Mexico), 7 = NM, 8 = OR, 9 = SD, 10 = WA, 11 = WY, 12 = BC

C57 = **LatSource**, which is the latitude given in the FHX file or publication

C58 = **LongSource**, which is the longitude given in the FHX file or publication

C59 = **Latitude**, which is the latitude converted to decimal degrees

C60 = **Longitude**, which is the longitude converted to decimal degrees

C61 = **UTM_E**, which is the UTM easting coordinate in meters, if given in the FHX file or publication

C62 = **UTM_N**, which is the UTM northing coordinate in meters, if given in the FHX file or publication

C63 = **Datum**, which is the datum, if given in the FHX file or publication

THIS LAST COLUMN IS WHETHER THIS CASE IS A CALIBRATION OR PREDICTION

C64 = **CalPred**, Calibration or Prediction
