# Supplementary material for: Restoring and managing low-severity fire in dry-forest landscapes of the western USA
Source: PLoS One. 2017 Feb 15;12(2):e0172288. doi: 10.1371/journal.pone.0172288 (PMC5310858; doi:10.1371/journal.pone.0172288)
Supplement: S1 Table — (PDF) [file pone.0172288.s005.pdf]

S1 Table. Authors, locations, and values for CFI and ITFI estimators and PMFI/FR in the 96-case calibration dataset. Refer to S2 Table for references for all citations.

Row number and Author(s)	Area	Sites	Area (ha)	No.†	Mean CFI (years)‡				Weib. Mean ITFI	PMFI/FR (years)§		
					Meth	All	10%	25%		Meth	YrsRec	YrsTot
1 Allen (1989)	N NM	Frijoles Canyon	6600	75	2	3.1	7.2	11.6		5	23.1	*30.9*
2 Arabas et al. (2006)	C OR	Lava Cast Forest	1100	85	3	4.8	9.8	24.8	37.0	4	39.9	83.3
3 Baisan and Swetnam (1990)	SE AZ	Mica Mountain	1600	44¶	3	3.1	5.6	7.4	12.5	4	12.6	15.0
4 Baker (2006)	N AZ	Powell, Fire Point, Rainbow Plateau	2566	119	1	6.9		13.1		1		45.1
5 Beaty and Taylor (2001)	N CA	Combined study areas	427	56	2	7.6		14.2		1		28.2
6 Beaty and Taylor (2001)	N CA	North-facing	105	8	2	33.8		33.8		1		42.5
7 Beaty and Taylor (2001)	N CA	Northern headwaters	102	15	2	16.3		26.1		1		27.2
8 Beaty and Taylor (2001)	N CA	South-facing	134	19	2	11.5		13.5		1		17.4
9 Beaty and Taylor (2001)	N CA	Southern headwaters	87	14	2	19.1		21.6		1		37.2
10 Bekker and Taylor (2001)	N CA	White fir-Jeffrey pine	537	18	2	5.5				1		21.5
11 Bekker and Taylor (2001)	N CA	White fir-Sugar pine	335	9	2	11.0				1		33.7
12 Bigio (2013)	SW CO	Haflin Canyon	600	19	3	14.2	16.8	26.4	42.1	4	34.3	50.8
13 Bigio (2013)	SW CO	Steven's Canyon	1000	28	3	12.8	15.3	22.2	37.6	4	44.0	74.0
14 Bigio et al. (2010)	SW CO	Vallecito Country Market	100	17	3	14.3	16.7	20.0	27.8	4	28.3	32.6
15 Bork (1984)	C OR	Cabin Lake	97	31	2	24.0				2		79.0
16 Bork (1984)	C OR	Lookout Mountain	97	48	2	15.0				2		77.0
17 Bork (1984)	C OR	Pringle Butte	97	35	2	11.0				2		31.0
18 Brown and Sieg (1996)	W SD	Jewel Cave East	150#	16	3	23.0	23.0	24.9	33.6	4	40.5	40.5

19 Brown and Wu (2005)	SW CO	Archuleta Mesa	307	52	3	10.0	15.9	16.9	23.1	4		32.1
20 Brown et al. (2008)	W SD	Mount Rushmore	517	29	2	16.0		17.0		1		30.0
21 Danzer (1998)	SE AZ	Pat Scott Peak	100	29	3	3.2	5.2	9.1	13.1	4	14.6	17.7
22 Dieterich (1983)	NE AZ	Thomas Creek	166	21	3	3.3	5.4	11.4	17.3	4	13.4	22.1
23 Dieterich and Hibbert (1990)	N AZ	Battle Flat	87	10	3	1.9	1.9	3.4	6.3	4	6.6	7.2
24 Donnegan et al. (2001)	C CO	Badger Mountain	100	55	3	9.0			92.8	4	81.6	94.1
25 Donnegan et al. (2001)	C CO	China Wall	100	9	3	27.5	27.5			4	102.0	138.3
26 Donnegan et al. (2001)	C CO	Salt Creek	100	9	3	23.6	23.6	55.2	89.1	4	74.1	106.7
27 Everett (2003)	S CA	Big Pine Flat	275	38	2	5.7				1	244.7	*327.2*
28 Everett (2003)	S CA	Black Mountain	270	33	2	5.2	17.9			1	201.5	*269.4*
29 Everett et al. (2000)	C WA	Mud Creek	12757	490	2	2.7				1		11.0
30 Everett et al. (2000)	C WA	Nile Creek	3240	233	2	2.6				1		12.2
31 Farris (2009)	S AZ	Mica Mountain	2780	405	2	2.2	9.2	25.5		1		26.8
32 Farris et al. (2013)	N AZ	Centennial Forest	100	612	2	1.8	2.7	5.4		3	9.0	*12.0*
33 Farris et al. (2013)	S AZ	Mica Mountain	2780	405	2	2.1	6.2	8.0		3	9.4	*12.6*
34 Farris et al. (2013)	N NM	Monument Canyon	256	197	2	2.7	5.7	7.3		3	10.7	*14.3*
35 Fiegener (2002)	S CA	Teakettle	90	61	2	4.0	6.7			1	37.3	*49.9*
36 Fiegener (2002)	S CA	Teakettle	90	61	2	4.0	6.7			3	56.3	*75.3*
37 Fulé et al. (1997)	N AZ	Camp Navajo	700	52	3	3.7	4.6	6.5	13.0	4	12.7	19.0
38 Fulé et al. (2003a)	N AZ	Galahad Point	410	31	3	4.0	4.7	6.8	11.3	4	10.0	12.5
39 Fulé et al. (2003b)	N AZ	Fire Point	135	39	3	3.7	4.9	6.4	11.3	4	10.2	13.6
40 Fulé et al. (2003b)	N AZ	Grandview	810	44	3	7.1	7.4	9.3	14.9	4	15.4	17.9
41 Fulé et al. (2003b)	N AZ	Powell Plateau	315	46	3	3.3	4.5	9.6	13.7	4	13.0	15.4

42 Fulé et al. (2003b)	N AZ	Rainbow Plateau	225	34	3	4.0	5.3	7.8	14.1	4	13.0	18.0
43 Fulé et al. (2003b)	N AZ	Swamp Ridge	270	30	3	5.3	6.9	8.8	14.6	4	14.1	17.1
44 Fulé et al. (2003b); Dugan and Baker (2014, 2015)	N AZ	Grandview	644	44	3	7.1	7.4	9.3	18.4	1		25.7
45 Fulé et al. (2009)	SW CO	Lower Middle Mountain	150	20	3	12.9	19.1	32.3	30.5	4	46.9	46.8
46 Grissino-Mayer et al. (2004)	SW CO	Benson Creek	200	23	3	7.9	7.9	9.4	14.9	4	13.4	20.7
47 Grissino-Mayer et al. (2004)	SW CO	Five Pine Canyon	200	15	3	6.8	6.8	11.0	16.0	4	12.9	15.8
48 Grissino-Mayer et al. (2004)	SW CO	Monument	200	18	3	20.6	22.6	25.1	33.2	4	27.3	37.5
49 Grissino-Mayer et al. (2004)	SW CO	Plateau	150	21	3	6.2	7.8	8.7	15.9	4	11.6	15.2
50 Grissino-Mayer et al. (2004)	SW CO	Smoothing Iron	200	23	3	13.3	16.5	21.5	25.7	4	22.2	29.0
51 Grissino-Mayer et al. (2004)	SW CO	Taylor Creek	100	16	3	18.7	21.8	21.8	26.7	4	23.6	29.2
52 Grissino-Mayer et al. (2004)	SW CO	Turkey Springs	200	28	3	10.8	10.8	13.3	21.1	4	14.4	26.4
53 Heinlein et al. (2005)	N AZ	San Francisco Peaks East	160	18	3	5.2	5.5	9.9	17.3	4	18.1	23.2
54 Heinlein et al. (2005)	N AZ	San Francisco Peaks West	160	16	3	5.4	5.4	9.1	15.5	4	16.2	20.6
55 Heyerdahl (1997)	NE OR	Baker City	3812	114	3	3.4	6.8	10.6	18.1	1		15.3
56 Heyerdahl (1997)	NE OR	Baker City	3812	114	3	3.4	6.8	10.6	18.1	4	16.5	22.7
57 Heyerdahl (1997)	NE OR	Dugout	8585	215	3	3.1	8.4	11.8	21.4	1		15.3
58 Heyerdahl (1997)	NE OR	Dugout	8585	215	3	3.1	8.4	11.8	21.4	4	17.3	24.8
59 Heyerdahl (1997)	NE OR	Imnaha	2095	110	3	5.0	11.1	19.9	33.8	1		28.4
60 Heyerdahl (1997)	NE OR	Imnaha	2095	110	3	5.0	11.1	19.9	33.8	4	24.1	37.5
61 Heyerdahl (1997)	NE OR	Tucannon	2002	86	3	5.7	9.8	26.2	39.8	1		30.5
62 Heyerdahl (1997)	NE OR	Tucannon	2002	86	3	5.7	9.8	26.2	39.8	4	27.5	41.4
63 Heyerdahl et al. (2008)	W MT	McCormick Creek	148	17	3	11.1	11.1	12.2	18.0	4	15.4	19.4
64 Heyerdahl et al. (2012)	S BC	Middle Stein River Valley	1105	155	3	3.5	8.2	14.7	27.9	4	21.0	40.5

65 Heyerdahl, no publication	C OR	Green Ridge	866	92	3	7.8	10.9	13.2	34.6	4	19.0	43.0
66 Heyerdahl, no publication	C OR	Lytle Creek	766	134	3	5.6	7.3	9.8	26.7	4	12.8	37.6
67 Heyerdahl, no publication	C OR	McKay Creek	782	115	3	7.9	8.8	10.1	24.5	4	15.6	35.3
68 Huffman et al. (2015)	N AZ	Mogollon Rim	1135	133	2	2.0	3.0	8.5		1	14.4	*19.3*
69 Jones (2005)	W MT	Lubrecht	379	46	3	9.0	10.5	14.4	23.3	4	19.9	27.4
70 Kernan and Hessl (2010)	E WA	Entiat	15708	469	2	3.3		7.0		1		13.1
71 Kernan and Hessl (2010)	E WA	Nile Creek	4033	232	2	3.3		10.0		1		17.0
72 Kernan and Hessl (2010)	E WA	South Deep	10809	151	2	9.3		26.0		1		45.3
73 Kernan and Hessl (2010)	E WA	Swauk	12644	665	2	1.8		14.0		1		15.8
74 Margolis and Balmat (2009)	N NM	Dry Mixed Conifer	1200	65	3	12.4	20.2	31.6	49.5	4	52.6	74.7
75 Margolis and Balmat (2009)	N NM	Ponderosa Pine	1600	76	3	4.3	9.1	20.8	25.8	4	26.5	39.8
76 Scholl and Taylor (2010)	C CA	Tuolomne Meadows	2125	209	2	1.2	2.4	10.7		3		13.0
77 Shapiro-Miller et al. (2007)	W ID	Powderhouse: 1924-1986	611	74	2	31.5				1		44.5
78 Shapiro-Miller et al. (2007)	W ID	Powderhouse: 1924-1986	611	74	2	31.5				4	37.2	38.9
79 Shapiro-Miller et al. (2007)	W ID	Powderhouse: 1739-1889	611	74	3	6.8	10.0	18.8	23.9	4	19.2	33.0
80 Taylor (2000)	N CA	Jeffrey Pine	473	36	2	4.9				1		24.5
81 Taylor (2000)	N CA	Jeffrey Pine-White Fir	868	22	2	7.5				1		31.3
82 Taylor and Skinner (1998)	N CA	1626-1849	1570	204	2					2		19.0
83 Taylor and Skinner (1998)	N CA	1850-1904	1570	204	2					2		12.3
84 Taylor and Skinner (1998)	N CA	1905-1992	1570	204	2					2		25.5
85 Taylor and Skinner (2003)	N CA	Hayfork: 1628-1849	2325	329	3	5.2	5.2	27.0	22.9	2		20.0
86 Taylor and Skinner (2003)	N CA	Hayfork: 1850-1904	2325	329	3	10.3	10.3	10.3		2		18.0
87 Touchan et al. (1996)	N NM	Clear Creek Campground	130	20	2	5.6	8.2	13.1	15.6	4	16.5	17.9

88 Touchan et al. (1996)	N NM	Gallina Mesa	285	25	2	5.1	8.0	11.5	18.5	4	20.2	24.7
89 Veblen et al. (2000)	N CO	BM6	89	11	3	103.0	103.0	103.0	80.9	4	100.0	100.0
90 Veblen et al. (2000)	N CO	BM10	119	7	3	40.0	40.0	63.0		4	67.9	112.6
91 Veblen et al. (2000)	N CO	BM15	105	37	3	8.0	10.1	17.4	30.1	4	27.0	44.9
92 Veblen et al. (2000)	N CO	BM18	95	21	3	51.3	51.3	68.3	79.0	4	77.5	103.5
93 Veblen et al. (2000)	N CO	BM22	148	11	3	47.0	47.0	47.0		4	49.8	61.9
94 Veblen et al. (2000)	N CO	BM23	164	12	3	28.8	29.6	29.6	63.6	4	53.4	80.3
95 Wright (1996); Wright and Agee (2004)	C WA	Teanaway Demonstration Area	30000	92	3	2.0	5.2	14.2	16.4	1		26.0
96 Wright (1996); Wright and Agee (2004)	C WA	Teanaway Demonstration Area	30000	92	3	2.0	5.2	14.2	16.4	4	16.0	20.2

Notes

† Number of fire-scarred trees sampled.

‡ Fire interval methods (**Meth**) include: 1. Author estimate from digital spatial fire records, 2. Author estimate in text, a table, or a graph, 3. My estimate from author's FHX file using FHAES

§ Fire rotation methods (**Meth**) include, in decreasing order of expected accuracy: 1. Author estimate from fire-year maps, fire atlases, or digital spatial fire records, 2. My estimate from area burned in an author table or graph, 3. Author estimate from the ratio method, 4. My estimate from the ratio method and author's FHX file using FHAES, 5. My estimate from the ratio method using author's data in a table or interpolated from a graph. **YrsTot** is fire rotation, in years, from a ratio method based on percentage of total sample trees or plots. **YrsRec** is fire rotation, in years, from a ratio method based on percentage of recorder trees. Where fire rotation-YrsTot was not available, I estimated it using the correction equation in Table 3 of the main text, and the estimate is shown in italics, but this estimate was not used in the regression analysis, only to provide a prediction estimate for the 342-case dataset.

¶ Baisan and Swetnam (1990) reported that 50 of 54 sampled fire-scarred trees could be cross-dated, but data from only 44 were in the IMPD dataset.

# This is my estimate from enclosing the fire-scarred trees (Brown and Sieg 1996 Figure 1) with a minimum rectangle.
