# Supplementary material for: Restoring and managing low-severity fire in dry-forest landscapes of the western USA
Source: PLoS One. 2017 Feb 15;12(2):e0172288. doi: 10.1371/journal.pone.0172288 (PMC5310858; doi:10.1371/journal.pone.0172288)
Supplement: S2 Table — (PDF) [file pone.0172288.s006.pdf]

S2 Table. Authors, sites, the Weibull mean ITFI estimate, and the calibrated or predicted PMFI/FR for the merged 342-site dataset.

State/Author(s)†	Sites	State	Weibull Mean ITFI (years)	Calibrated or predicted	Calibrated/ Predicted PMFI/FR (years)
ARIZONA					
Dieterich and Hibbert (1990)	Battle Flat	AZ	6.31	Calibrated	7.20
Kaib and Swetnam, no publ.	Mt. Ord	AZ	8.72	Predicted	10.60
Dieterich (1980)	Chimney Springs	AZ	8.80	Predicted	10.70
Swetnam and Baisan (1996)	Walnut Canyon	AZ	8.81	Predicted	10.71
Swetnam et al. (2001)	Palisades	AZ	9.21	Predicted	11.20
Farris et al. (2013)	Centennial Forest	AZ	-	Predicted	12.03
Fulé et al. (2003a)	Galahad Point	AZ	11.26	Calibrated	12.50
Seklecki et al. (1996)	Rustler Park	AZ	10.32	Predicted	12.55
Farris et al. (2013)	Mica Mountain	AZ	-	Predicted	12.57
Baisan et al. (1998)	Rose Canyon Lower	AZ	10.80	Predicted	13.13
Danzer (1998)	Sawmill Canyon	AZ	10.92	Predicted	13.28
Fulé et al. (2003b)	Fire Point	AZ	11.25	Calibrated	13.60
Baisan and Swetnam (1990)	Mica Mountain	AZ	12.48	Calibrated	15.00
Baisan et al. (1998)	Mount Lemmon	AZ	12.36	Predicted	15.03
Fulé et al. (2003b)	Powell Plateau	AZ	13.68	Calibrated	15.40
Baisan et al. (1998)	Rose Canyon Upper	AZ	12.74	Predicted	15.49
Swetnam and Baisan (1996)	Josephine Saddle	AZ	12.90	Predicted	15.69
Baisan et al. (1998)	Rose Canyon East	AZ	13.40	Predicted	16.29
Fulé et al. (2003b)	Swamp Ridge	AZ	14.56	Calibrated	17.10
Danzer (1998)	Pat Scott Peak	AZ	13.06	Calibrated	17.70
Fulé et al. (2003b)	Grandview	AZ	14.89	Calibrated	17.90
Fulé et al. (2003b)	Rainbow Plateau	AZ	14.10	Calibrated	18.00
Fulé et al. (1997)	Camp Navajo	AZ	13.02	Calibrated	19.00
Huffman et al. (2015)	Mogollon Rim	AZ	-	Predicted	19.25
Heinlein et al. (2005)	San Francisco Peaks West	AZ	15.50	Calibrated	20.60
Dieterick (1983)	Thomas Creek	AZ	17.31	Calibrated	22.10
Heinlein et al. (2005)	San Francisco Peaks East	AZ	17.32	Calibrated	23.20
Fulé et al. (2003b; Dugan and Baker (2014)	Grandview	AZ	18.40	Calibrated	25.70
CALIFORNIA					
Caprio and Swetnam (1995)	Ash Peak Ridge	CA	7.04	Predicted	8.56

Taylor and Skinner (1998)	Thompson Ridge: 1850-1904	CA	-	Calibrated	12.30
Scholl and Taylor (2010)	Tuolomne River	CA	-	Calibrated	13.00
Beaty and Taylor (2001)	South-facing	CA	-	Calibrated	17.40
Caprio and Swetnam (1995)	Bobcat Point Pine	CA	15.09	Predicted	18.35
Taylor and Skinner (1998)	Thompson Ridge: 1626-1849	CA	-	Calibrated	19.00
Taylor and Skinner (2003)	Hayfork: 1628-1849	CA	22.86	Calibrated	20.00
Bekker and Taylor (2001)	White fir-Jeffrey pine	CA	-	Calibrated	21.50
Caprio and Swetnam (1995)	High Sierra Ridge Pine	CA	18.75	Predicted	22.80
Taylor (2000)	Prospect Peak: Jeffrey Pine	CA	-	Calibrated	24.50
Beaty and Taylor (2001)	Northern headwaters	CA	-	Calibrated	27.20
Beaty and Taylor (2001)	Combined study areas	CA	-	Calibrated	28.20
Taylor (2000)	Prospect Peak: Jeffrey Pine-White fir	CA	-	Calibrated	31.30
Bekker and Taylor (2001)	White fir-Sugar pine	CA	-	Calibrated	33.70
Beaty and Taylor (2001)	Southern headwaters	CA	-	Calibrated	37.20
Swetnam et al., no publication	Buck Rock Flat	CA	32.11	Predicted	39.05
Beaty and Taylor (2001)	North-facing	CA	-	Calibrated	42.50
Fiegener (2002)	Teakettle	CA	-	Predicted	49.87
Fiegener (2002)	Teakettle	CA	-	Predicted	75.31
Everett (2003)	Black Mountain	CA	-	Predicted	269.41
Everett (2003)	Big Pine Flat	CA	-	Predicted	327.16
COLORADO					
Grissino-Mayer et al. (2004)	Plateau	CO	15.86	Calibrated	15.20
Grissino-Mayer et al. (2004)	Five Pine Canyon	CO	15.96	Calibrated	15.80
Veblen et al. (2000)	BM34	CO	14.31	Predicted	17.40
Brown and Wu (2005)	Archuleta Mesa Plot A05	CO	15.19	Predicted	18.47
Grissino-Mayer et al. (2004)	Benson Creek	CO	14.86	Calibrated	20.70
Grissino-Mayer et al. (2004)	Hermosa Creek	CO	20.97	Predicted	25.50
Brown and Wu (2005)	Archuleta Mesa Plot A1	CO	21.49	Predicted	26.13
Grissino-Mayer et al. (2004)	Turkey Springs	CO	21.14	Calibrated	26.40
Veblen et al. (2000)	BM31	CO	23.68	Predicted	28.79
Grissino-Mayer et al. (2004)	Smoothing Iron	CO	25.74	Calibrated	29.00
Grissino-Mayer et al. (2004)	Taylor Creek	CO	26.72	Calibrated	29.20
Brown and Shepperd (2001)	Wet Mountains South	CO	25.85	Predicted	31.43
Brown and Wu (2005)	Archuleta Mesa	CO	23.13	Calibrated	32.10
Veblen et al. (2000)	BM14	CO	26.79	Predicted	32.58

Brown and Shepperd (2001)	M Kaufmanns Cabin	CO	26.80	Predicted	32.59
Bigio et al. (2010)	Vallecito Country Market	CO	27.75	Calibrated	32.60
Grissino-Mayer et al. (2004)	Monument	CO	33.16	Calibrated	37.50
Bigio (2013)	Marina Basin	CO	30.95	Predicted	37.64
Grissino-Mayer et al. (2004)	Burnette Canyon	CO	33.83	Predicted	41.14
Brown and Shepperd (2001)	Black Mountain	CO	35.28	Predicted	42.90
Brown and Wu (2005)	Archuleta Mesa Plot AA1	CO	36.00	Predicted	43.78
Veblen et al. (2000)	BM15	CO	30.12	Calibrated	44.92
Fulé et al. (2009)	Lower Middle Mountain	CO	30.53	Calibrated	46.80
Veblen et al. (2000)	BM28	CO	39.34	Predicted	47.84
Brown and Wu (2005)	Archuleta Mesa Plot B2	CO	40.02	Predicted	48.66
Veblen et al. (2000)	BM24	CO	40.26	Predicted	48.96
Brown and Wu (2005)	Archuleta Mesa Plot C2	CO	41.52	Predicted	50.49
Bigio et al. (2010)	Haflin Canyon	CO	42.11	Calibrated	50.80
Veblen et al. (2000)	BM11	CO	42.27	Predicted	51.40
Brown and Shepperd (2001)	Manitou Demo Plot	CO	42.34	Predicted	51.49
Veblen et al. (2000)	BM8	CO	47.23	Predicted	57.43
Veblen et al. (2000)	BM9	CO	48.61	Predicted	59.11
Brown and Shepperd (2001)	Mica Mine	CO	49.71	Predicted	60.45
Brown and Wu (2005)	Archuleta Mesa Plot AA15	CO	50.41	Predicted	61.30
Veblen et al. (2000)	BM22	CO	-	Calibrated	61.90
Brown and Shepperd (2001)	Parachute Hill	CO	51.75	Predicted	62.93
Veblen et al. (2000)	BM20	CO	53.57	Predicted	65.14
Veblen et al. (2000)	BM32	CO	58.07	Predicted	70.61
Brown et al. (2000)	Hot Creek	CO	58.44	Predicted	71.06
Brown and Wu (2005)	Archuleta Mesa Plot B3	CO	58.97	Predicted	71.71
Veblen et al. (2000)	BM13	CO	59.80	Predicted	72.72
Bigio (2013)	Steven’s Canyon	CO	37.56	Calibrated	74.00
Brown and Wu (2005)	Archuleta Mesa Plot C5	CO	64.07	Predicted	77.91
Veblen et al. (2000)	BM23	CO	63.59	Calibrated	80.30
Brown and Shepperd (2001)	Left Hand Canyon	CO	67.75	Predicted	82.38
Veblen et al. (2000)	BM5	CO	71.75	Predicted	87.25
Veblen et al. (2000)	BM12	CO	72.73	Predicted	88.44
Brown and Wu (2005)	Archuleta Mesa Plot A15	CO	73.80	Predicted	89.74
Donnegan et al. (2001)	BSA Shortcut	CO	75.54	Predicted	91.86
Brown and Shepperd (2001)	Cheesman Lake South	CO	76.87	Predicted	93.47

Donnegan et al. (2001)	Badger Mountain	CO	92.84	Calibrated	94.10
Brown and Shepperd (2001)	Washout Gulch Burn	CO	77.56	Predicted	94.31
Brown and Shepperd (2001)	Cheesman Lake North	CO	78.62	Predicted	95.60
Veblen et al. (2000)	BM6	CO	80.88	Calibrated	100.00
Veblen et al. (2000)	BM18	CO	78.98	Calibrated	103.50
Brown and Shepperd (2001)	Old Tree Cluster	CO	86.85	Predicted	105.61
Donnegan et al. (2001)	Salt Creek	CO	89.07	Calibrated	106.70
Brown and Shepperd (2001)	Lone Pine	CO	88.59	Predicted	107.73
Veblen et al. (2000)	BM39	CO	88.81	Predicted	107.99
Veblen et al. (2000)	BM10	CO	-	Calibrated	112.60
Veblen et al. (2000)	BM21	CO	99.22	Predicted	120.65
Brown and Shepperd (2001)	Lone Pine Upper	CO	107.72	Predicted	130.99
Donnegan et al. (2001)	China Wall	CO	-	Calibrated	138.30
Veblen et al. (2000)	BM19	CO	114.59	Predicted	139.34
Veblen et al. (2000)	BM17	CO	143.99	Predicted	175.09
IDAHO					
Heyerdahl et al. (2008)	Warm Springs Ridge	ID	13.88	Predicted	16.88
Heyerdahl et al. (2008)	Bannock Creek	ID	13.98	Predicted	17.00
Heyerdahl et al. (2008)	Wash Creek	ID	15.98	Predicted	19.43
Heyerdahl et al. (2008)	Keating Ridge	ID	22.26	Predicted	27.07
Heyerdahl et al. (2008)	Cove Mountain	ID	25.53	Predicted	31.04
Shapiro-Miller et al. (2007)	Powderhouse	ID	23.89	Calibrated	32.95
Heyerdahl et al. (2008)	Lowman RNA	ID	30.73	Predicted	37.37
MONTANA					
Heyerdahl et al. (2008)	Sophie Lake	MT	10.90	Predicted	13.25
Heyerdahl et al. (2008)	Sheldon Flats	MT	11.05	Predicted	13.44
Heyerdahl et al. (2008)	Butler Creek	MT	12.22	Predicted	14.86
Heyerdahl et al. (2008)	Blue Mountain	MT	12.28	Predicted	14.93
Heyerdahl et al. (2008)	McCormick Creek	MT	18.00	Calibrated	19.40
Heyerdahl et al. (2008)	McMillan Mountain	MT	17.71	Predicted	21.54
Heyerdahl et al. (2008)	Corona Road	MT	19.25	Predicted	23.41
Heyerdahl et al. (2008)	Hunter Point	MT	19.84	Predicted	24.13
Heyerdahl et al. (2008)	Sheafman Creek	MT	21.06	Predicted	25.61
Jones (2005)	Lubrecht	MT	23.26	Calibrated	27.40
Heyerdahl et al. (2008)	Crane Lookout	MT	25.47	Predicted	30.97
Heyerdahl et al. (2008)	Sawmill Creek RNA	MT	27.00	Predicted	32.83

NEW MEXICO					
Brown et al. (2001)	Pines at Sunspot	NM	8.40	Predicted	10.21
Swetnam and Dieterich (1985)	Langstroth Mesa	NM	8.62	Predicted	10.48
Kaye and Swetnam (1999)	Lower San Andreas	NM	9.74	Predicted	11.84
Morino (1996)	Upper Fillmore West	NM	10.55	Predicted	12.83
Grissino-Mayer & Swetnam (1997)	Cerro Bandera North	NM	10.71	Predicted	13.02
Swetnam and Dieterich (1985)	Gilita Ridge	NM	10.81	Predicted	13.14
Swetnam and Dieterich (1985)	McKenna Park	NM	11.05	Predicted	13.44
Kaye and Swetnam (1999)	Lower Pine Spring	NM	11.56	Predicted	14.06
Farris et al. (2013)	Monument Canyon	NM	-	Predicted	14.31
Brown et al. (2001)	James Ridge	NM	12.11	Predicted	14.73
Swetnam et al., no publication	Cerro Balitas	NM	12.32	Predicted	14.98
Morino (1996)	Upper Fillmore Side Cany. 1	NM	12.48	Predicted	15.18
Kaye and Swetnam (1999)	Upper San Andreas	NM	13.02	Predicted	15.83
Grissino-Mayer & Swetnam (1997)	Cerro Bandera East	NM	13.10	Predicted	15.93
Morino (1996)	Snag Saddle	NM	14.14	Predicted	17.19
Baisan and Swetnam (1997)	Capilla Peak Campground	NM	14.45	Predicted	17.57
Morino (1996)	Fillmore Side Canyon 2	NM	14.69	Predicted	17.86
Touchan et al. (1996)	Clear Creek Campground	NM	15.61	Calibrated	17.90
Grissino-Mayer & Swetnam (1997)	Candelaria	NM	14.97	Predicted	18.20
Grissino-Mayer & Swetnam (1997)	La Marchanita	NM	14.97	Predicted	18.20
Kaye and Swetnam (1999)	Cherry Canyon	NM	15.23	Predicted	18.52
Swetnam et al. (2001)	Black Mountain	NM	15.91	Predicted	19.35
Baisan and Swetnam (1997)	Canon de Turrieta	NM	16.02	Predicted	19.48
Morino (1996)	Rock House Spring	NM	16.10	Predicted	19.58
Brown et al. (2001)	Monument Canyon	NM	16.17	Predicted	19.66
Morino (1996)	Narrows	NM	16.42	Predicted	19.97
Kaye and Swetnam (1999)	Upper Pine Spring	NM	18.11	Predicted	22.02
Morino (1996)	Fillmore Side Canyon	NM	18.20	Predicted	22.13
Morino (1996)	Ledge Site	NM	18.44	Predicted	22.42
Brown et al. (2001)	Monument Canyon Upper	NM	18.66	Predicted	22.69
Touchan et al. (1996)	Pajarito Mountain Ridge	NM	19.04	Predicted	23.15
Baisan and Swetnam (1997)	La Luz Trail	NM	20.29	Predicted	24.67
Touch an et al. (1996)	Gallina Mesa	NM	18.54	Calibrated	24.70
Swetnam and Baisan (1996)	Ice Canyon	NM	21.58	Predicted	26.24
Swetnam (1990)	Bear Wallow	NM	21.74	Predicted	26.44

Swetnam and Baisan (1996)	Continental Divide Peak	NM	21.86	Predicted	26.58
Grissino-Mayer & Swetnam (1997)	Cerro Rendija	NM	22.19	Predicted	26.98
Grissino-Mayer & Swetnam (1997)	Mesita Blanca	NM	23.15	Predicted	28.15
Grissino-Mayer & Swetnam (1997)	Lost Woman	NM	23.50	Predicted	28.58
Swetnam et al., no publication	Laguna Garule	NM	23.79	Predicted	28.93
Grissino-Mayer & Swetnam (1997)	Hoya de Cibola Lava Flow	NM	24.03	Predicted	29.22
Swetnam and Baisan (1996)	El Calderon	NM	24.54	Predicted	29.84
Allen (1989)	Frijoles Canyon	NM	-	Predicted	30.88
Brown et al. (2001)	Delworth	NM	25.73	Predicted	31.29
Brown et al. (2001)	Fir Campground	NM	25.85	Predicted	31.43
Brown et al. (2001)	Peake Canyon	NM	28.63	Predicted	34.81
Touchan et al. (1996)	Camp May East	NM	28.91	Predicted	35.15
Brown et al. (2001)	Cosmic Ray Obs	NM	29.79	Predicted	36.22
Swetnam and Baisan (1996)	Continental Divide Saddle	NM	29.81	Predicted	36.25
Brown et al. (2001)	Sunspot	NM	29.89	Predicted	36.35
Baisan et al., no publication	Bonita Canyon	NM	30.70	Predicted	37.33
Touchan et al. (1996)	Canada Bonita South	NM	31.53	Predicted	38.34
Margolis and Balmat (2009)	Santa Fe Watershed Ponderosa Pine	NM	25.78	Calibrated	39.80
Touchan et al. (1996)	Cerro Pedernal	NM	33.56	Predicted	40.81
Grissino-Mayer & Swetnam (1997)	Hidden Kipuka	NM	38.90	Predicted	47.30
Margolis and Balmat (2009)	Santa Fe Watershed Dry Mixed Conifer	NM	49.46	Calibrated	74.70
OREGON					
Heyerdahl (1997), Heyerdahl et al. (2001)	Baker City	OR	18.11	Calibrated	15.30
Heyerdahl (1997), Heyerdahl et al. (2001)	Dugout	OR	21.39	Calibrated	15.30
Maruoka (1994)	Spring Mountain (12)	OR	16.40	Predicted	19.94
Heyerdahl (1997), Heyerdahl et al. (2001)	Baker City	OR	18.11	Calibrated	22.70
Maruoka (1994)	Seed Orchard (4)	OR	18.69	Predicted	22.73
Maruoka (1994)	Widow's Creek (1)	OR	19.72	Predicted	23.98
Maruoka (1994)	East Camp Creek (5)	OR	20.17	Predicted	24.53
Heyerdahl (1997), Heyerdahl et al. (2001)	Dugout	OR	21.39	Calibrated	24.80
Maruoka (1994)	Smoothing Iron Ridge (15)	OR	21.93	Predicted	26.67
Maruoka (1994)	Little Bear Burn (7)	OR	23.19	Predicted	28.20

Heyerdahl (1997), Heyerdahl et al. (2001)	Imnaha	OR	33.82	Calibrated	28.40
Maruoka (1994)	Five Mile Creek (6)	OR	24.11	Predicted	29.32
Maruoka (1994)	West Myrtle Creek (8)	OR	24.67	Predicted	30.00
Bork (1984)	Pringle Butte	OR	-	Calibrated	31.00
Heyerdahl, no publication	McKay Creek	OR	24.47	Calibrated	35.30
Heyerdahl (1997), Heyerdahl et al. (2001)	Imnaha	OR	33.82	Calibrated	37.50
Heyerdahl, no publication	Lytle Creek	OR	26.70	Calibrated	37.57
Maruoka (1994)	Raddue (2)	OR	33.27	Predicted	40.46
Heyerdahl, no publication	Green Ridge	OR	34.62	Calibrated	42.96
Maruoka (1994)	Troy (14)	OR	36.84	Predicted	44.80
Maruoka (1994)	Dixie Butte (3)	OR	43.64	Predicted	53.07
Bork (1984)	Lookout Mountain	OR	-	Calibrated	77.00
Bork (1984)	Cabin Lake	OR	-	Calibrated	79.00
Arabas et al. (2006)	Lava Cast Forest	OR	37.00	Calibrated	83.25
SOUTH DAKOTA					
Brown and Sieg (1999)	Pigtail Bridge	SD	17.42	Predicted	21.18
Brown and Sieg (1999)	Wind Cave North	SD	19.44	Predicted	23.64
Wienk et al. (2004)	Badger Game Prod. Area	SD	22.24	Predicted	27.04
Brown et al. (2008)	Mount Rushmore	SD	-	Calibrated	30.00
Brown (2003, 2006)	Black Hills Plot 105	SD	26.30	Predicted	31.98
Brown (2003, 2006)	Black Hills Plot 111	SD	26.85	Predicted	32.65
Brown and Sieg (1996)	Jewel Cave South	SD	27.20	Predicted	33.08
Brown (2003, 2006)	Black Hills Plot 204	SD	27.89	Predicted	33.91
Brown (2003)	Bear Lodge Central	SD	28.87	Predicted	35.11
Brown (2003, 2006)	Black Hills Plot 210	SD	28.95	Predicted	35.20
Brown (2003)	Reynold's Prairie	SD	28.95	Predicted	35.20
Brown (2003, 2006)	Black Hills Plot 213	SD	30.05	Predicted	36.54
Brown and Sieg (1996)	Jewel Cave North	SD	30.08	Predicted	36.58
Brown and Sieg (1999)	Gobbler Ridge	SD	31.02	Predicted	37.72
Brown (2003, 2006)	Black Hills Plot 207	SD	32.25	Predicted	39.22
Brown (2003)	Riflepit Gulch West	SD	32.72	Predicted	39.79
Brown (2003, 2006)	Black Hills Plot 202	SD	32.72	Predicted	39.79
Brown (2003, 2006)	Black Hills Plot 109	SD	33.02	Predicted	40.15
Brown and Sieg (1996)	Jewel Cave East	SD	33.58	Calibrated	40.52
Brown (2003, 2006)	Black Hills Plot 209	SD	34.04	Predicted	41.39

Brown et al. (2000)	Upper Pine Mid-Basin	SD	34.78	Predicted	42.29
Brown (2003)	Black Hills Exp. Forest	SD	35.22	Predicted	42.83
Brown (2003, 2006)	Black Hills Plot 112	SD	36.02	Predicted	43.80
Brown and Sieg (1996)	Jewel Cave Central	SD	36.47	Predicted	44.35
Brown (2003)	Bear Lodge North	SD	37.75	Predicted	45.90
Brown (2003, 2006)	Black Hills Plot 205	SD	38.42	Predicted	46.72
Brown (2003, 2006)	Black Hills Plot 106	SD	38.84	Predicted	47.23
Brown (2003, 2006)	Black Hills Plot 208	SD	40.05	Predicted	48.70
Brown (2003, 2006)	Black Hills Plot 113	SD	40.11	Predicted	48.77
Brown (2003, 2006)	Black Hills Plot 114	SD	40.75	Predicted	49.55
Brown (2003, 2006)	Black Hills Plot 203	SD	41.21	Predicted	50.11
Brown (2003)	Riflepit Gulch East	SD	41.35	Predicted	50.28
Brown (2003)	Riflepit Gulch North	SD	42.56	Predicted	51.75
Brown (2003, 2006)	Black Hills Plot 101	SD	42.75	Predicted	51.98
Brown (2003, 2006)	Black Hills Plot 206	SD	44.86	Predicted	54.55
Brown (2003, 2006)	Black Hills Plot 201	SD	46.10	Predicted	56.06
Brown (2003, 2006)	Black Hills Plot 110	SD	46.16	Predicted	56.13
Brown (2003, 2006)	Black Hills Plot 108	SD	63.33	Predicted	77.01
Brown (2003, 2006)	Black Hills Plot 103	SD	36.95 §	Predicted	90.16
Brown (2003, 2006)	Black Hills Plot 104	SD	64.33 ¶	Predicted	158.70
WASHINGTON					
Everett et al. (2000)	Entiat Mud Creek overall	WA	-	Calibrated	11.00
Everett et al. (2000)	Nile Creek overall	WA	-	Calibrated	12.20
Kernan and Hessl (2010)	Entiat	WA	-	Calibrated	13.10
Everett et al. (2000)	Entiat Mud Creek 165	WA	11.92	Predicted	14.49
Everett et al. (2000)	Entiat Mud Creek 230	WA	11.98	Predicted	14.57
Everett et al. (2000)	Entiat Mud Creek 201	WA	12.41	Predicted	15.09
Everett et al. (2000)	Entiat Mud Creek 205	WA	12.50	Predicted	15.20
Kernan and Hessl (2010)	Swauk	WA	-	Calibrated	15.80
Everett et al. (2000)	Entiat Mud Creek 199	WA	13.08	Predicted	15.91
Everett et al. (2000)	Entiat Mud Creek 202	WA	13.70	Predicted	16.66
Kernan and Hessl (2010)	Nile Creek	WA	-	Calibrated	17.00
Everett et al. (2000)	Nile Creek 10	WA	14.21	Predicted	17.28
Everett et al. (2000)	Entiat Mud Creek 196	WA	14.29	Predicted	17.38
Everett et al. (2000)	Entiat Mud Creek 207	WA	15.07	Predicted	18.33
Everett et al. (2000)	Entiat Mud Creek 203	WA	15.32	Predicted	18.63

Everett et al. (2000)	Nile Creek 5	WA	15.46	Predicted	18.80
Everett et al. (2000)	Quartzite 1	WA	15.54	Predicted	18.90
Everett et al. (2000)	Entiat Mud Creek 208	WA	16.02	Predicted	19.48
Everett et al. (2000)	Entiat Mud Creek 167	WA	16.17	Predicted	19.66
Everett et al. (2000)	Frosty 8	WA	16.38	Predicted	19.92
Wright (1996); Wright and Agee (2004)	Teanaway Demonstration Area	WA	16.43	Calibrated	20.20
Everett et al. (2000)	Quartzite 8	WA	16.73	Predicted	20.34
Everett et al. (2000)	Entiat Mud Creek 200	WA	16.84	Predicted	20.48
Everett et al. (2000)	Quartzite 6	WA	16.93	Predicted	20.59
Everett et al. (2000)	Nile Creek 3	WA	17.22	Predicted	20.94
Everett et al. (2000)	Quartzite 3	WA	17.40	Predicted	21.16
Everett et al. (2000)	Frosty 7	WA	17.47	Predicted	21.24
Everett et al. (2000)	Nile Creek 9	WA	17.66	Predicted	21.47
Everett et al. (2000)	Entiat Mud Creek 206	WA	17.78	Predicted	21.62
Everett et al. (2000)	Quartzite 2	WA	18.10	Predicted	22.01
Everett et al. (2000)	Nile Creek 4	WA	18.14	Predicted	22.06
Everett et al. (2000)	Frosty 4	WA	18.51	Predicted	22.51
Everett et al. (2000)	Frosty 3	WA	18.68	Predicted	22.71
Everett et al. (2000)	Frosty 2	WA	19.00	Predicted	23.10
Everett et al. (2000)	South Deep 1	WA	19.01	Predicted	23.12
Everett et al. (2000)	Quartzite 4	WA	19.17	Predicted	23.31
Everett et al. (2000)	Nile Creek 8	WA	19.36	Predicted	23.54
Everett et al. (2000)	Quartzite 5	WA	19.53	Predicted	23.75
Everett et al. (2000)	Entiat Mud Creek 204	WA	19.76	Predicted	24.03
Everett et al. (2000)	Nile Creek 2	WA	20.28	Predicted	24.66
Everett et al. (2000)	Nile Creek 6	WA	20.36	Predicted	24.76
Everett et al. (2000)	Frosty 1	WA	20.38	Predicted	24.78
Everett et al. (2000)	Nile Creek 1	WA	21.09	Predicted	25.65
Wright (1996); Wright and Agee (2004)	Teanaway Demonstration Area	WA	16.43	Calibrated	26.00
Everett et al. (2000)	Entiat Mud Creek 211	WA	21.71	Predicted	26.40
Everett et al. (2000)	Twenty Mile 3	WA	22.85	Predicted	27.79
Everett et al. (2000)	Frosty 6	WA	23.20	Predicted	28.21
Everett et al. (2000)	Quartzite 7	WA	24.28	Predicted	29.52
Everett et al. (2000)	Nile Creek 11	WA	24.65	Predicted	29.97

Heyerdahl (1997), Heyerdahl et al. (2001)	Tucannon	WA	39.80	Calibrated	30.50
Everett et al. (2000)	Twenty Mile 4	WA	25.60	Predicted	31.13
Everett et al. (2000)	Twenty Mile 1	WA	26.03	Predicted	31.65
Everett et al. (2000)	Frosty 5	WA	26.10	Predicted	31.74
Everett et al. (2000)	Twenty Mile 2	WA	26.68	Predicted	32.44
Everett et al. (2000)	Nile Creek 7	WA	29.34	Predicted	35.68
Everett et al. (2000)	Twenty Mile 6	WA	29.54	Predicted	35.92
Everett et al. (2000)	Twenty Mile 7	WA	31.24	Predicted	37.99
Everett et al. (2000)	South Deep 6	WA	31.58	Predicted	38.40
Everett et al. (2000)	Twenty Mile 8	WA	32.42	Predicted	39.42
Everett et al. (2000)	Twenty Mile 13	WA	33.75	Predicted	41.04
Heyerdahl (1997), Heyerdahl et al. (2001)	Tucannon	WA	39.80	Calibrated	41.40
Kernan and Hessl (2010)	South Deep	WA	-	Calibrated	45.30
Everett et al. (2000)	Twenty Mile 9	WA	40.17	Predicted	48.85
Everett et al. (2000)	Twenty Mile 12	WA	40.20	Predicted	48.88
Everett et al. (2000)	South Deep 7	WA	41.91	Predicted	50.96
Everett et al. (2000)	South Deep 3	WA	43.33	Predicted	52.69
Everett et al. (2000)	South Deep 5	WA	44.13	Predicted	53.66
Everett et al. (2000)	South Deep 9	WA	47.74	Predicted	58.05
Everett et al. (2000)	Twenty Mile 10	WA	48.96	Predicted	59.54
Everett et al. (2000)	South Deep 11a	WA	51.65	Predicted	62.81
Everett et al. (2000)	South Deep 11b	WA	51.65	Predicted	62.81
Everett et al. (2000)	South Deep 12	WA	51.65	Predicted	62.81
Everett et al. (2000)	South Deep 14	WA	51.82	Predicted	63.01
Everett et al. (2000)	South Deep 4	WA	53.37	Predicted	64.90
Everett et al. (2000)	South Deep 10	WA	55.09	Predicted	66.99
Everett et al. (2000)	Twenty Mile 11	WA	67.38	Predicted	81.93
WYOMING					
Brown (2003)	Cold Springs Creek	WY	24.63	Predicted	29.95
Brown et al. (2000)	Ashenfelder Lower	WY	45.88	Predicted	55.79
Brown et al. (2000)	Ashenfelder Upper	WY	45.88	Predicted	55.79
MEXICO					
Skinner et al. (2008)	PINO (San Pedro Martir)	MX	19.04	Predicted	23.15
Skinner et al. (2008)	BLAN (San Pedro Martir)	MX	22.18	Predicted	26.97
Skinner et al. (2008)	PYRA (San Pedro Martir)	MX	24.89	Predicted	30.27

Skinner et al. (2008)	WEST (San Pedro Martir)	MX	25.02	Predicted	30.42
Skinner et al. (2008)	TASA (San Pedro Martir)	MX	26.55	Predicted	32.28
Skinner et al. (2008)	VALL (San Pedro Martir)	MX	26.67	Predicted	32.43
Skinner et al. (2008)	PUER (San Pedro Martir)	MX	28.07	Predicted	34.13
Skinner et al. (2008)	CORO (San Pedro Martir)	MX	30.94	Predicted	37.62
Skinner et al. (2008)	AZUL (San Pedro Martir)	MX	55.99	Predicted	68.08
CANADA-BRITISH COLUMBIA					
Heyerdahl et al. (2012)	Middle Stein River Valley	BC	27.93	Calibrated	40.49

Notes

† Observations are in increasing order of calibrated/predicted PMFI/FR within each state

‡ Missing observations in this column occur because some calibration cases did not have an FHX file and did not report this statistic in the publication.

§ Mean ITFI could not be estimated, but mean CFI-all could be, is reported here, and was used to estimate PMFI/FR

¶ Mean ITFI could not be estimated, but mean CFI-10% could be, is reported here, and was used to estimate PMFI/FR

Literature Cited

Allen, C. D. 1989. Changes in the landscape of the Jemez Mountains, New Mexico. Ph.D. dissertation. University of California, Berkeley.

Arabas, K. B., K. S. Hadley, and E. R. Larson. 2006. Fire history of a naturally fragmented landscape in central Oregon. Canadian Journal of Forest Research 36:1108-1120.

Baisan, C. H., and T. W. Swetnam. 1990. Fire history on a desert mountain range: Rincon Mountain Wilderness, Arizona, U.S.A. Canadian Journal of Forest Research 20:1559-1569.

Baisan, C. H., and Swetnam, T. W. 1997. Interactions of fire regimes and land use in the central Rio Grande Valley. USDA Forest Service Research Paper RM-RP-330, Rocky Mountain Forest and Range Experiment Station, Fort Collins, Colorado.

Baisan, C. H., K. A. Morino, H. D. Grissino-Mayer, and T. W. Swetnam. 1998. Fire history in ponderosa pine and mixed-conifer forests of the Catalina Mountains. Final Report, Laboratory of Tree-Ring Research, University of Arizona, Tucson.

Baker, W. L. 2006. Fire history in ponderosa pine landscapes of Grand Canyon National park: is it reliable enough for management and restoration? International Journal of Wildland Fire 15:433-437.

Beaty, R. M., and A. H. Taylor. 2001. Spatial and temporal variation of fire regimes in a mixed conifer forest landscape, southern Cascades, California, USA. Journal of Biogeography 28:955-966.

Bekker, M. F., and A. H. Taylor. 2001. Gradient analysis of fire regimes in montane forests of the southern Cascade Range, Thousand Lakes Wilderness, California, USA. Plant Ecology 155:15-28.

Bigio, E. R. 2013. Late Holocene fire and climate history of the western San Juan Mountains, Colorado: Results from alluvial stratigraphy and tree-ring methods. PhD dissertation, University of Arizona, Tucson.

Bigio, E., T. W. Swetnam, and C. H. Baisan. 2010. A comparison and integration of tree-ring and alluvial records of fire history at the Missionary Ridge Fire, Durango, Colorado, USA. The Holocene 20:1047-1061.

Bork, J. L. 1984. Fire history in three vegetation types of the eastern side of the Oregon

Cascades. Ph.D. dissertation. Oregon State University, Corvallis.
Brown, P. M. 2003. Fire, climate, and forest structure in ponderosa pine forests of the Black Hills. PhD dissertation, Colorado State University, Fort Collins.
Brown, P. M. 2006. Climate effects on fire regimes and tree recruitment in Black Hills ponderosa pine forests. Ecology 87:2500-2510.
Brown, P. M., and W. D. Shepperd. 2001. Fire history and fire climatology along a 5° gradient in latitude in Colorado and Wyoming, USA. Palaeobotanist 50:133-140.
Brown, P. M., and C. H. Sieg. 1996. Fire history in interior ponderosa pine communities of the Black Hills, South Dakota, USA. International Journal of Wildland Fire 6:97-105.
Brown, P. M., and C. H. Sieg. 1999. Historical variability in fire at the ponderosa pine - northern Great Plains prairie ecotone, southeastern Black Hills, South Dakota. Ecoscience 6:539-547.
Brown, P. M., C. L. Wienk, and A. J. Symstad. 2008. Fire and forest history at Mount Rushmore. Ecological Applications 18:1984-1999.
Brown, P. M., and R. Wu. 2005. Climate and disturbance forcing of episodic tree recruitment in a southwestern ponderosa pine landscape. Ecology 86:3030-3038.
Brown, P. M., M. G. Ryan, and T. G. Andrews. 2000. Historical surface fire frequency in ponderosa pine stands in Research Natural Areas, central Rocky Mountains and Black Hills, USA. Natural Areas Journal 20:133-139.
Brown, P. M., M. W. Kaye, L. S. Huckaby, and C. H. Baisan. 2001. Fire history along environmental gradients in the Sacramento Mountains, New Mexico: Influences of local patterns and regional processes. Ecoscience 8:115-126.
Caprio, A. C., and T. W. Swetnam. 1995. Historic fire regimes along an elevational gradient on the west slope of the Sierra Nevada, California. Pages 173-179 In: J. K. Brown, R. W. Mutch, C. W. Spoon, and R. H. Wakimoto, editors. Proceedings: symposium on fire in wilderness and park management. USDA Forest Service General Technical Report INT-GTR-320, Intermountain Research Station, Ogden, Utah.
Danzer, S. R. 1998. Fire history and stand structure in the Huachuca Mountains of southeastern Arizona. Master's. University of Arizona, Tucson.
Dieterich, J. H. 1980. Chimney Spring forest fire history. USDA Forest Service Research Paper RM-220, Rocky Mountain Forest and Range Experiment Station, Fort Collins, Colorado.
Dieterich, J. H. 1983. Fire history of southwestern mixed conifer: a case study. Forest Ecology and Management 6:13-31.
Dieterich, J. H., and A. R. Hibbert. 1990. Fire history in a small ponderosa pine stand surrounded by chaparral. Page 168-173 In: Krammes, J. S., editor. Effects of fire management of southwestern natural resources. Gen. Tech. Rep. RM-GTR-191. Fort Collins, CO: U.S. Department of Agriculture, Forest Service, Rocky Mountain Forest and Range Experiment Station.
Donnegan, J. A., T. T. Veblen, and J. S. Sibold. 2001. Climatic and human influences on fire history in Pike National Forest, central Colorado. Canadian Journal of Forest Research 31:1526-1539.
Dugan, A. J., and W. L. Baker. 2014. Modern calibration and historical testing of small-area, fire-interval reconstruction methods. International Journal of Wildland Fire 23:58-68.
Dugan, A. J., and W. L. Baker. 2015. Sequentially contingent fires, droughts and pluvials structured a historical dry forest landscape and suggest future contingencies. Journal of Vegetation Science 26:697-710.
Everett, R. G. 2003. Grid-based fire-scar dendrochronology and vegetation sampling in the mixed-conifer forests of the San Bernardino and San Jacinto Mountains of southern California. PhD dissertation, University of California, Riverside, California.
Everett, R. L., R. Schellhaas, D. Keenum, D. Spurbeck, and P. Ohlson. 2000. Fire history in the

ponderosa pine/Douglas-fir forests on the east slope of the Washington Cascades. Forest Ecology and Management 129:207-225.
Farris, C. A. 2009. Comparison of multiple methods for inferring fire history in a Sky Island mountain range. Ph.D. dissertation. University of Arizona, Tucson, Arizona, USA.
Farris, C. A., C. H. Baisan, D. A. Falk, M. L. Van Horne, and Fulé. 2013. A comparison of targeted and systematic fire-scar sampling for estimating historical fire frequency in south-western ponderosa pine forests. International Journal of Wildland Fire 22:1021-1033.
Fiegener, R. P. 2002. The influence of sampling intensity on the fire history of the Teakettle Experimental Forest, Sierra Nevada, California: small fire detection, the composite fire chronology, and fire interval calculation. University of California, Davis.
Fulé, P. Z., W. W. Covington, and M. M. Moore. 1997. Determining reference conditions for ecosystem management of southwestern ponderosa pine forests. Ecological Applications 7:895-908.
Fulé, P. Z., J. E. Crouse, T. A. Heinlein, M. M. Moore, W. W. Covington, and G. Verkamp. 2003a. Mixed-severity fire regime in a high-elevation forest of Grand Canyon, Arizona, USA. Landscape Ecology 18:465-486.
Fulé, P. Z., T. A. Heinlein, W. W. Covington, and M. M. Moore. 2003b. Assessing fire regimes on Grand Canyon landscapes with fire-scar and fire-record data. International Journal of Wildland Fire 12:129-145.
Fulé, P. Z., J. E. Korb, and R. Wu. 2009. Changes in forest structure of a mixed conifer forest, southwestern Colorado, USA. Forest Ecology and Management 258:1200-1210.
Grissino-Mayer, H. D. 1995. Tree-ring reconstructions of climate and fire history at El Malpais National Monument, New Mexico. PhD dissertation, University of Arizona, Tucson.
Grissino-Mayer, H. D., and T. W. Swetnam. 1997. Multi-century history of wildfire in the ponderosa pine forests of El Malpais National Monument. Bulletin 156, New Mexico Bureau of Mines and Mineral Resources, Socorro, New Mexico.
Grissino-Mayer, H. D., W. H. Romme, M. L. Floyd, and D. D. Hanna. 2004. Climatic and human influences on fire regimes of the southern San Juan Mountains, Colorado, USA. Ecology 85:1708-1724.
Heinlein, T. A., M. M. Moore, P. Z. Fulé, and W. W. Covington. 2005. Fire history and stand structure of two ponderosa pine-mixed conifer sites: San Francisco Peaks, Arizona, USA. International Journal of Wildland Fire 14: 307-320.
Heyerdahl, E. K. 1997. Spatial and temporal variation in historical fire regimes of the Blue Mountains, Oregon and Washington: the influence of climate. Ph.D. dissertation. University of Washington, Seattle.
Heyerdahl, E. K., L. B. Brubaker, and J. K. Agee. 2001. Spatial controls of historical fire regimes: a multiscale example from the interior West, USA. Ecology 82:660-678.
Heyerdahl, E. K., K. Lertzman, and C. M. Wong. 2012. Mixed-severity fire regimes in dry forests of southern interior British Columbia, Canada. Canadian Journal of Forest Research 42:88-98.
Heyerdahl, E. K., P. Morgan, and J. F. Riser II. 2008. Crossdated fire histories (1650 to 1900) from ponderosa pine-dominated forests of Idaho and western Montana. USDA Forest Service General Technical Report RMRS-GTR-214WWW, Rocky Mountain Research Station, Fort Collins, Colorado.
Huffman, D. W., T. J. Zegler, and P. Z. Fulé. 2015. Fire history of a mixed conifer forest on the Mogollon Rim, northern Arizona, USA. International Journal of Wildland Fire 24:680-689.
Jones, L.A. 2005. A surface fire history of the Lubrecht Experimental Forest. Missoula, MT. Undergraduate thesis, University of Montana, Missoula.
Kaye, M. W., and T. W. Swetnam. 1999. An assessment of fire, climate, and Apache history in

the Sacramento Mountains, New Mexico. Physical Geography 20:305-330.
Kernan, J. T., and A. E. Hessl. 2010. Spatially heterogeneous estimates of fire frequency in ponderosa pine forests of Washington, USA. Fire Ecology 6:117-135.
Margolis, E. Q., and J. Balmat. 2009. Fire history and fire–climate relationships along a fire regime gradient in the Santa Fe Municipal Watershed, NM, USA. Forest Ecology and Management 258:2416-2430.
Maruoka, K. R. 1994. Fire history of *Pseudotsuga menziesii* and *Abies grandis* stands in the Blue Mountains of Oregon and Washington. Master's thesis, University of Washington, Seattle, Washington.
Morino, K. A. 1996. Reconstruction and interpretation of historical patterns of fire occurrence in the Organ Mountains, New Mexico. M.S. thesis, University of Arizona, Tucson.
Scholl, A. E., and A. H. Taylor. 2010. Fire regimes, forest change, and self-organization in an old-growth mixed-conifer forest, Yosemite National Park, USA. Ecological Applications 20:362-380.
Seklecki, M. T., H. D. Grissino-Mayer, and T. W. Swetnam. 1996. Fire history and the possible role of Apache-set fires in the Chiricahua Mountains of southeastern Arizona. Pages 238-246 In: Ffolliott, P. F., L. F. DeBano, M. B. Baker, Jr., G. J. Gottfried, G. Solis-Garza, C. B. Edminster, D. G. Neary, L. S. Allen, and R. H. Hamre, editors. Effects of fire on Madrean province ecosystems: a symposium proceedings. USDA Forest Service General Technical Report RM-GTR-289, Rocky Mountain Forest and Range Experiment Station, Fort Collins, Colorado.
Shapiro-Miller, L. B., E. K. Heyerdahl, and P. Morgan. 2007. Comparison of fire scars, fire atlases, and satellite data in the northwestern United States. Canadian Journal of Forest Research 37:1933-1943.
Skinner, C. N., J. H. Burk, M. G. Barbour, E. Franco-Vizcaino, and S. L. Stephens. 2008. Influences of climate on fire regimes in montane forests of north-western Mexico. Journal of Biogeography 35:1436-1451.
Swetnam, T. W. 1990. Fire history and climate in the southwestern United States. Pages 6-17 In: J. S. Krammes, editor. Effects of fire management of southwestern natural resources. USDA Forest Service General Technical Report RM-191, Rocky Mountain Research Station, Fort Collins, Colorado.
Swetnam, T. W., and C. H. Baisan. 1996. Historical fire regime patterns in the southwestern United States since AD 1700. Pages 11-32 In: C. D. Allen, editor. Fire effects in southwestern forests, proceedings of the second La Mesa fire symposium. USDA Forest Service General Technical Report RM-GTR-286, Rocky Mountain Forest and Range Experiment Station, Fort Collins, Colorado.
Swetnam, T. W., C. H. Baisan, and J. M. Kaib. 2001. Forest fire histories of the Sky Islands of *La Frontera*. Pages 95-124 In: G. L. Webster, and C. J. Bahre, editors. Changing plant life of La Frontera. University of New Mexico Press, Albuquerque.
Swetnam. T. W., and J. H. Dieterich. 1985. Fire history of ponderosa pine forests in the Gila Wilderness, New Mexico. Pages 390-397 In: Proceedings–Symposium and workshop on wilderness fire. USDA Forest Service General Technical Report INT-182, Intermountain Forest and Range Experiment Station, Ogden, Utah.
Taylor, A. H. 2000. Fire regimes and forest changes in mid and upper montane forests of the southern Cascades, Lassen Volcanic National Park, California, U.S.A. Journal of Biogeography 27:87-104.
Taylor, A. H., and C. N. Skinner. 1998. Fire history and landscape dynamics in a late-successional reserve, Klamath Mountains, California, USA. Forest Ecology and Management 111:285-301.

Taylor, A. H., and C. N. Skinner. 2003. Spatial patterns and controls on historical fire regimes and forest structure in the Klamath Mountains. Ecological Applications 13:704-719.

Touchan, R., C. D. Allen, and T. W. Swetnam. 1996. Fire history and climatic patterns in ponderosa pine and mixed-conifer forests of the Jemez Mountains, northern New Mexico. Pages 33-46 In: C. D. Allen, editor. Fire effects in southwestern forests: proceedings of the second La Mesa fire symposium. USDA Forest Service General Technical Report RM-GTR-286, Rocky Mountain Forest and Range Experiment Station, Fort Collins, Colorado.

Veblen, T. T., T. Kitzberger, and J. Donnegan. 2000. Climatic and human influences on fire regimes in ponderosa pine forests in the Colorado Front Range. Ecological Applications 10:1178-1195.

Wienk, C. L., C. H. Sieg, and G. R. McPherson. 2004. Evaluating the role of cutting treatments, fire and soil seed banks in an experimental framework in ponderosa pine forests of the Black Hills, South Dakota. Forest Ecology and Management 192:375-393.

Wright, C. S. 1996. Fire history of the Teanaway River Drainage, Washington. M.S. Thesis, University of Washington, Seattle.

Wright, C. S., and J. K. Agee. 2004. Fire and vegetation history in the eastern Cascade Mountains, Washington. Ecological Applications 14:443-459.
