# Supplementary material for: Restoring and managing low-severity fire in dry-forest landscapes of the western USA
Source: PLoS One. 2017 Feb 15;12(2):e0172288. doi: 10.1371/journal.pone.0172288 (PMC5310858; doi:10.1371/journal.pone.0172288)
Supplement: S1 Text — (PDF) [file pone.0172288.s007.pdf]

S1 Text. Why CFIs and ITFIs underestimate PMFI/FR

Since both mean composite fire interval (CFI) and mean individual tree fire interval (ITFI) typically underestimate the population mean fire interval or fire rotation (PMFI/FR), it is logical to infer that both methods are missing longer intervals because of biased estimates (Table 1 main text). Compositing alone could explain nearly the whole bias in CFI measures, as explained below, but ITFI measures are not composites and are still biased, although less so. The most likely explanation for bias in ITFI measures, and also a contributor to bias in CFI measures, as estimators of PMFI/FR, is targeted sampling. These potential sources of bias are reviewed in detail here.

Compositing overcompensates, destroys long fire intervals, and restriction rules do not remedy this

Scarring fraction, compositing, and widespread over-compensation

The purpose of compositing is to compensate for the incomplete scar record on individual trees, since trees can often resist scarring even if burned (Baker and Dugan 2013). Scarring fraction (SF) is the fraction of burned live trees that survive a fire but receive a scar. Studies of SF are few (e.g., Collins and Stephens 2007, Stephens et al. 2010). A study of 16 fires in ponderosa pine forests in northern Arizona found a mean SF of 0.375, ranging from 0.121 to 0.728 across 52 plot samples (Baker and Dugan 2013).

Given a particular SF, how many trees must be sampled or composited to compensate for SF < 1.0 (Baker and Dugan 2013)? The minimum is to have a sample size that has a high probability of recording each fire on at least one scarred tree. The probability, P, of at least one tree scarring in a sample of n live trees, for a scarring fraction, SF, is given by:

$$P = 1.0 - (1.0 - \mathrm{SF})^{n} \qquad (1)$$

and the corresponding estimate of n, for a particular SF, is given by:

$$n = \log (1.0 - P) / \log (1.0 - \mathrm{SF}) \qquad (2)$$

The necessary sample sizes to achieve a probability ≥ 0.95 or 0.99 of detecting a fire are modest, typically < 20 trees, whether scarred or not, to detect fires with SF < 0.25 (Figure A).

However, this equation does not adjust for scar healing. Scars can, but do not always, heal from the sides and disappear under new bark unless subsequent fires occur (Baker and Dugan 2013). However, Fiegener (2002) examined over 8,000 stumps and snags in a Sierran mixed-conifer forest and found only 2% with scars. An empirical study of scar healing after fires in northern Arizona ponderosa pine forests

Figure A. Scarring fraction and its effect on the sample size needed to have either a 0.95 or 0.99 probability of scarring at least one tree.

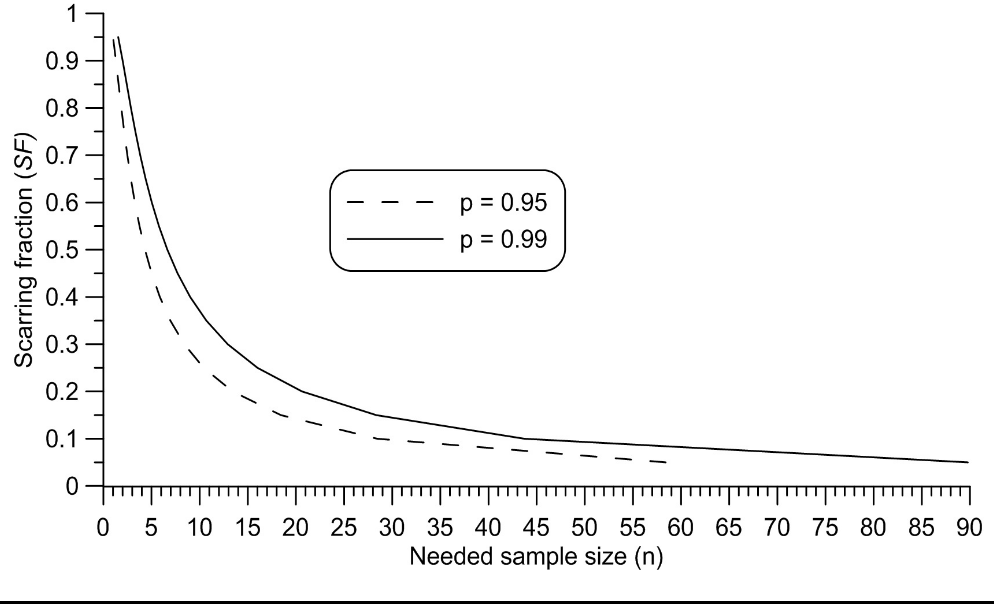

showed that larger initial scars have longer expected healing times and subsequent fires increased healing times of all scars (Baker and Dugan 2013). Healing rates from this study can be used to estimate the needed sample size to find at least one unhealed scar for a fire after 100 years, or another time since fire, using an equation developed from forests differing in time since fire:

$$\text{Effective mean SF} = \text{Initial SF} \times \exp(-0.0125 \times \text{Time since fire}) \qquad (3)$$

For example, if expected mean SF is 0.400, then six sample trees would likely ($P = 0.95$) contain at least one scar (using Eq. 2 or Figure A) from a fire burned recently. In contrast, the effective mean SF if that same fire had burned 100 years ago, and scars had healed since then, would be 0.115, from Eq. 3, thus requiring a sample of about 26 trees each > 100 years old (Eq. 1, Figure A). Similarly, a lower SF of 0.200 would require 51 trees > 100 years old for a fire 100 years ago. These calculations suggest that sufficient trees to detect fires in historical landscapes could likely be obtained from unlogged areas that are on the order of about 1 ha in area or even less.

In contrast, most compositing is from areas far too large for the area and number of sample trees usually needed to compensate for SF < 1.0. In the merged dataset of 342 sites, only 262 reported area sampled. Of those, only 32 (12.2%) were from areas < 1.0 ha (Figure B). One concern is whether SF rates estimated in this study are higher than they would have been in historical forests, because fire exclusion increased fuel loads in modern forests, likely increasing SF. Some effect is likely, but the effect would not change the general pattern of widespread overcompensation. First, if a preceding fire occurred within 30 years, then SF was reduced from a mean of 0.393 to 0.324, only an 18% reduction, in the Baker and Dugan (2013) study. This would have a minor effect of increasing the number of needed sample trees from 26 trees to 31 trees, having almost no effect on the widespread pattern of over-compensation evident in Figure B. Second, even in the extreme case of a 0.05 mean SF in historical forests, assuming a historical fire rotation of < 10 years (Stephens et al. 2010), only 208 trees, whether scarred or not, would be needed after 100 years to achieve a probability ≥0.95 of detecting a fire. This could be obtained in most historical dry forests in < 2-3 ha. Even at this extreme level of SF, only 18.7% of the 262 sites were from areas < 3 ha, thus 81.3% of studies were over-compensating.

Figure B. Number of cases (n = 262 total) by sample area (ha). Many cases did not report area. The first bin is from 0-1 ha, the remainder are 25-ha wide, and the last bin is for areas > 250 ha.

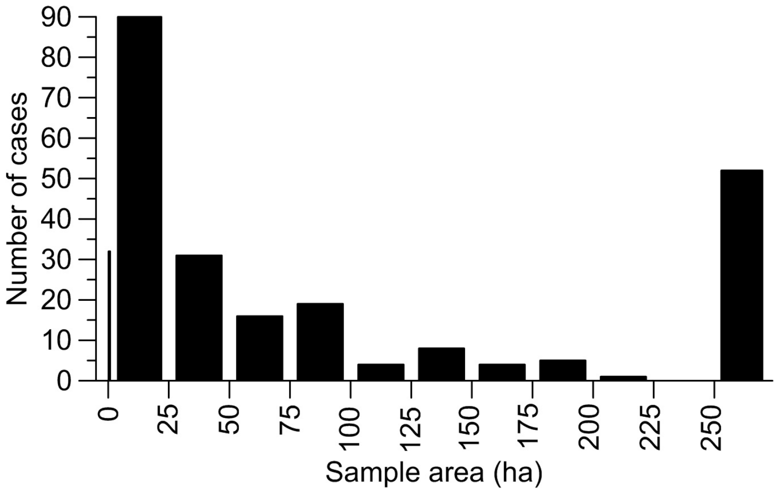

This over-compensation particularly biases CFI estimates toward values that are too short, since mean CFI declines as sample area or number of sample trees increases (Arno and Petersen 1983, Baker and Ehle 2001, Everett 2003, Kou and Baker 2006a, b). ITFI and FR estimates, in contrast, do not systematically decline with larger samples, and may even become more precise. Compositing records across an area or number of trees that is too large could explain why CFI estimates are too short relative to FRs, but cannot explain why ITFI estimates, which do not use compositing, are also too short.

Compositing not only over-compensates, but also destroys long fire intervals that are real

Compositing is a processing step, separate from finding and collecting an adequate sample. Several methods can be used to process sample data, including calculating mean ITFI (Dugan and Baker 2014), or estimating FR, thus the compositing step is not essential. How does the compositing step contribute error if used to estimate PMFI/FR? Most fires are small and only a few are large (Baker and Ehle 2001). When a composite list is created, and intervals are calculated among fires in the list, each small fire year counts the same as a large fire year. Even though some compositing might offset incomplete evidence, at the same time it destroys other evidence. Longer fire-free intervals that are real occur in unburned parts of landscapes adjacent to where small fires occurred, and some long intervals that are false because scarring is incomplete also occur. However, all these long intervals, whether real or false, are erased across the whole sample area when a composite list is created, rather than disappearing only in the area where a small fire occurred (Figure C). Since longer intervals, some of which are real, are all lost to compositing, this in part explains why mean CFI underestimates PMFI/FR.

CFI restriction rules are ad hoc, inconsistent, and likely insufficient in excluding small fires

Some suggest that there is only a problem with mean CFI and its use if it is presented without omitting spot fires: "...this becomes a problem only if the fire chronology is presented with all fires, even the smaller spot fires, and is interpreted by the reader as if the chronology indicates how often the entire stand burned" (Stephens et al. 2003 p. 1091).

Restriction rules are traditionally applied to filter out fires, like spot fires, that are small, using the number of fire scars or the percentage of total scarred trees that record a fire year (e.g., 10%, 25%). However, no way is known to objectively identify a spot fire or other small fire that should be omitted, since fire-size distributions are typically nearly linear on a log-log plot and have no natural breaks (Kou and Baker 2006b). Also, distributions vary in slope among forest types and environments (Kou and Baker 2006b), so imposing a particular filter (e.g., 25%) has varying effects. This means that restriction rules are ad hoc

Figure C. How compositing destroys long fire intervals that are real: (a) a landscape that burned (shaded area) in each of three fire years between 1760-1860, and (b) the actual mean fire intervals (center panel), the mean composite fire interval for the landscape (left panel) calculated from a composite list of fires (1760, 1784, 1812, ending in 1860), thus three intervals in 100 years = 33.3 years, and the PMFI (right panel), which is the grand mean of the actual mean fire intervals (1500 years/16 = 93.8 years). Note that the reason that the mean CFI underestimates the PMFI is that compositing treats the 1784 and 1812 fire years as though they burned the same land area as the 1760 fire year, which also eliminates the 100.0-year fire intervals that occurred over most of the landscape.

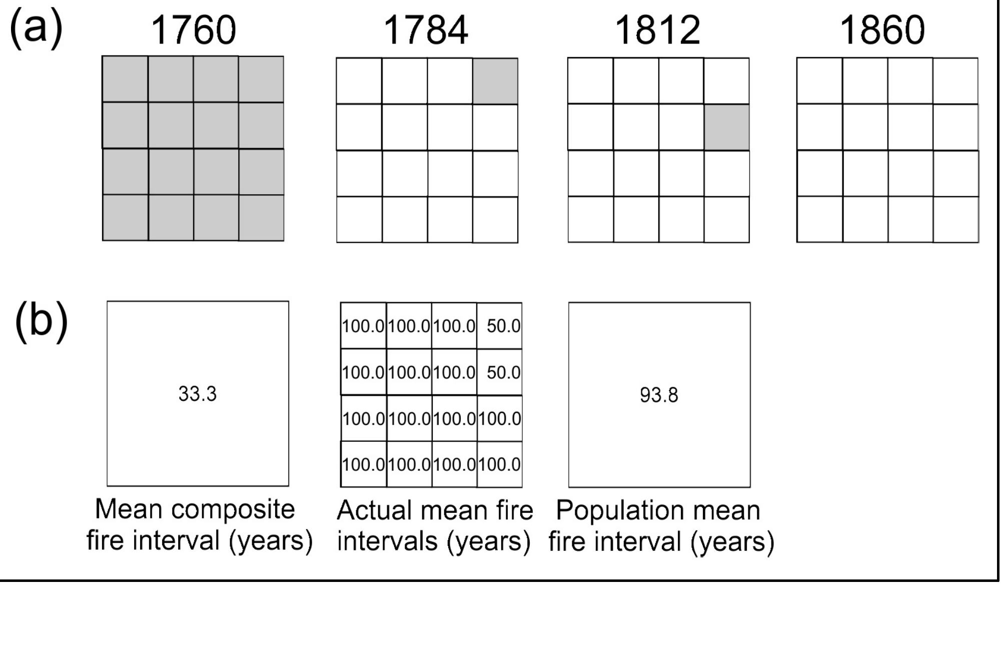

and inherently inconsistent in their effects.

Moreover, 10% and 25% filters that are typically applied, may be insufficient to limit the fires that should be included in a composite list, if the goal is that mean intervals between fires in the list estimate the PMFI/FR. In a spatial reconstruction of fire sizes in dry forests, Farris et al. (2010) found that 414 total fires occurred in their study area from 1937-2000, but only 21 fires (5.1% of total fires) accounted for 97% of total burned area. This suggests a restriction rule would have to exclude 95% of fires to limit a composite to the fire years that account for most of the total burned area, which would more likely accurately estimate PMFI/FR. Together, the ad hoc, inconsistent, and insufficient extent of traditional restriction rules in part explain why mean CFI underestimates PMFI/FR.

Censoring incomplete fire intervals leaves out long intervals in both CFI and ITFI estimates

Fire-history data contain incomplete intervals at the beginning and end of a period of record unless those periods begin and end with fires (Polakow and Dunne 1999). Incomplete intervals can be included or omitted ("censored") in analysis of fire-interval data (Polakow and Dunne 1999). Censoring (i.e., using only scar-to-scar intervals) biases both mean CFI and ITFI by omitting incomplete intervals at the beginning or end of a tree's record. Incomplete fire intervals occur on most trees, but longer intervals have more chance, than shorter intervals, of appearing as incomplete intervals, indicated by no scars or one scar on a tree (Kou and Baker 2006a). Simulation has shown that in a landscape subject to low-severity fires at modest intervals (e.g., 50 years), actual intervals at some locations may be several times longer (Kou and Baker 2006a) and even up to an order of magnitude longer than the mean interval (Parsons et al. 2007). These are real intervals that occur by chance, not an artifact of incomplete scarring. Censoring is biased against these expected long fire intervals and leads to estimates of PMFI/FR that are too short and have reduced variability, since longer intervals are omitted (Kou and Baker 2006a). These effects from censoring were also found in two studies in Mediterranean shrublands, in which censoring reduced the scale parameter (indicator of length of fire intervals) of a Weibull fire-interval distribution and also reduced estimated variability in fire intervals (Polakow and Dunne 1999, Moritz et al. 2009).

These censoring effects have ecological implications in dry forests subject to periodic fires, since most composited fire-scar records, which are traditionally censored, lack evidence of the long intervals needed for tree regeneration and survival of fire-intolerant species. We suggested that the interval before the first fire scar (origin-to-scar interval--OS) on individual trees may record the fire-free period needed for trees to successfully regenerate (Baker and Ehle 2001), since both wide-area and local processes producing long intervals should be recorded as OS intervals. Mean OS intervals are, in fact, usually much longer than mean scar-to-scar intervals in the same stands, and many are sufficiently long to allow tree regeneration (Baker and Ehle 2001). Mean OS intervals in ponderosa pine forests were 51 years in the Black Hills (Brown et al. 2008), 55.4 years in Rocky Mountain National Park (Baker and Ehle 2003), 81 years across five studies (Baker and Ehle 2001), and 101.5 years in one case in northern Arizona (Van Horne and Fulé 2006). Arguments can be made for and against including the OS interval in CFI estimates (e.g., Baker and Ehle 2003, Van Horne and Fulé 2006, Stephens et al. 2010). However, long intervals that are real do occur and are directly censored by traditional use of only scar-to-scar intervals in CFI and ITFI estimates, contributing to underestimation of PMFI/FR by CFI measures.

Targeted sampling likely a significant source of underestimates of PMFI/FR by ITFI, as well as by CFI

Why researchers target fire-history evidence and why it remains a concern for estimating PMFI/FR

Researchers target fire-history evidence to increase the length of record and maximize the data obtained with minimal physical effort and damage to trees (Farris et al. 2013). If only 50 scarred trees can be sampled, more fire years per scarred tree and a longer mean length of record will nearly always be obtained from 50 trees selected by targeting than from a random sample.

Unfortunately, targeting fire-history evidence at the scale of individual trees, sampling areas, and landscapes produces biased estimates of fire history (Lorimer 1985, Johnson and Gutsell 1994, Baker

and Ehle 2001). The consequences are generally that estimates of historical PMFI/FR are too short and fire-severity is underestimated. The magnitude of targeting and its effects is now better known. Targeting remains common in fire-history studies, as illustrated in Table A, which shows that targeting of individual trees, particularly multi-scarred trees and old trees, was widespread, almost universal for multi-scarred trees, and almost 1/3 of studies placed study plots where there were concentrations of scarred trees and old trees.

Table A. Percentage of 342 sites in which various types of targeting sampling were used.

**Targeting type and measures**	**Yes**	**No**	**No explanation**
1. Target trees to get best information or longest record of fires?			
Number of cases	114	68	160
Percentage of yes/no (%)	62.6	37.4	-
2. Target multi-scarred trees?			
Number of cases	235	15	92
Percentage of yes/no (%)	94.0	6.0	-
3. Target clusters of scarred trees?			
Number of cases	37	9	296
Percentage of yes/no (%)	80.4	19.6	
4. Target scars on dead wood?			
Number of cases	270	27	45
Percentage of yes/no (%)	90.9	9.1	
5. Target tree species thought to better record fire			
Number of cases	12	13	317
Percentage of yes/no (%)	48.0	52.0	
6. Target plot locations in old forests and concentrations of scars			
Number of cases	73	153	116
Percentage of yes/no (%)	32.3	67.7	
7. Target study areas in old forests and concentrations of scars			
Number of cases	18	168	156
Percentage of yes/no (%)	9.7	90.3	

Specific studies of some of these types of targeting are now available (Baker and Ehle 2003, Van Horne and Fulé 2006, Kou and Baker 2006a, Brown et al. 2008, Farris et al. 2010, 2013), but the most significant types are less studied. Studies whose findings supported targeted sampling (e.g., Van Horne

and Fulé 2006) for some purposes did not study using targeted sampling for estimating PMFI/FR, the focus here, thus targeted sampling has not been supported for this purpose.

Targeting individual trees

Targeting individual trees typically includes a bias component and a non-random sampling component. The bias component is from omitting trees with no scars or one scar and preferentially or exclusively using trees with multiple scars. The non-random sampling component comes from purposely choosing particular multi-scarred trees rather than randomly sampling them.

Significant bias is likely from omission of trees with no or single scars, which are traditionally omitted because only scar-to-scar intervals provide estimates of complete fire intervals. However, no scars or single scars on a tree may be false, because fires do not scar every tree that burns, but no or single-scarred trees also include real but incomplete fire intervals. No or single scars that represent real, incomplete long fire intervals are more likely where fire intervals also are longer (Kou and Baker 2006a). More long intervals and more of the length of long fire intervals are inherently present on unscarred or single-scarred trees, assuming tree ages are similar to those of multiple-scarred trees. Since longer fire intervals are more likely to be omitted by individual-tree targeting, all types of estimators (i.e., CFI, ITFI) from multi-scarred trees are biased toward being too short (Kou and Baker 2006a). FR estimates are also biased toward being too short if only multi-scarred trees are sampled, because trees with no scar or one scar can indicate places where a fire did not burn, and these omissions inflate area burned for that fire year, and shorten the estimated FR.

How does targeting trees with more than one scar (multi-scarred trees, recorder trees, and open-scarred trees) lead to CFI and ITFI values that underestimate PMFI/FR? Trees have visible, open scars because they are the trees that have had fires often enough to prevent healing. We found that the time for a fire scar to heal had a median of 38 years and was <100 years for 89% of scars (Baker and Dugan 2013). Longer fire intervals, that are real, have a high probability of not being selected by targeting trees with > 1 scar, because longer intervals often are expressed as no scars or one scar. Of course, long intervals can be an artifact of incomplete scarring, so that including all of them would lead to bias, but excluding all of them does too. Targeting trees with > 1 scar omits trees most likely to have long real fire intervals and selects trees with short fire intervals.

The substantial numerical dominance of unscarred and single-scarred trees in dry forests suggests omission of longer real fire intervals by individual-tree targeting of trees with > 1 scar could be among the most significant sources of bias in CFI estimates and possibly the main source of bias in ITFI estimates. In a sample of 906 pre-EuroAmerican trees we collected on 8 transects in northern Arizona, near Flagstaff and in Grand Canyon National Park, 779 trees had no scar (86%), 111 had one scar (12%), and only 16 trees had two or more scars (2%). In a mixed-conifer forest in the western Sierra, 98% of nearly 8,000 stumps and snags examined for scars did not have any scars, only 13 (0.2%) had one scar, and 48 (0.6%) had two or more scars (Fiegener 2002). Multi-scarred trees are rare in modern landscapes.

The magnitude of effects of omitting trees with no or one scar is unstudied, but within the set of multi-scarred trees with ≥2 scars, the effect of restricting fire history to increasing levels of multiple-scarring was studied (Fiegener 2002). To gauge how relevant this study is to multi-scarred sets of trees actually used in fire histories, I analyzed the number of scars per tree found by studies in the merged dataset, although data were available for only 324 cases. First, I calculated mean number of scarred trees, over each site's sample period, which is less than the total number of sample trees, since trees usually each cover only part of the sample period. Then, I calculated mean number of scars per scarred tree as total number of scars/mean number of scarred trees from the summary table for the FHX file in FHAES.

A histogram of mean scars per scarred tree had a mean of 8.47 scars/sample tree and a median of 7.61 scars/sample tree (Figure D). Fiegener (2002) found that restriction to ≥3 scars reduced ITFI from 17.4 years to 16.8 years (to 96.6%). This is above the minimum of 0.46 scars/tree in the distribution (Figure D). Restriction to ≥4 scars, just above the 1st quartile in the distribution, reduced ITFI to 15.8

Figure D. Histogram of the ratio of mean scars per sample tree in the 342-case merged dataset, and parameters of the distribution. Mean scars could not be calculated for 18 cases.

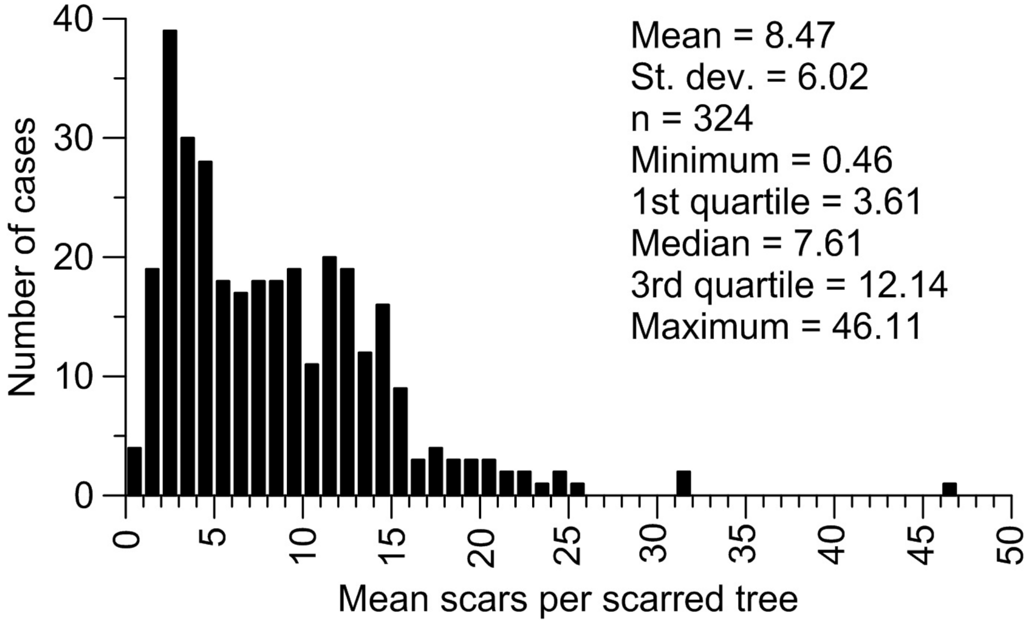

years (to 90.8%), restriction to ≥7 scars, just below the median, reduced ITFI to 14.6 years (to 83.9%), and restriction to ≥10 scars, below the 3rd quartile, reduced ITFI to 13.2 years (to 75.9%). These responses show that the more scars on a multi-scarred tree, the shorter is the mean ITFI estimate.

Roughly the median level of multi-scar targeting (≥7 scars), which reduced ITFI to 83.9%, or by 16.1%, closely matches the -16.64% bias in Weibull mean ITFI relative to PMFI/FR-total scarred trees/plots (Main text--Table 1). Other ITFI measures have biases of -2.71 to -29.71 (Main text-Table 1), so the close match with the Weibull Mean ITFI could possibly be a coincidence. Mean CFI-10% scarred also declined from 6.7 years to 5.7 years (85.1%) with restriction to ≥3 scars, but fluctuated or increased with higher levels of restriction (Fiegener 2002). Thus, the response of ITFI to targeting multi-scarred trees could explain much of why ITFI underestimates PMFI/FR, but the response of CFI estimators was inconsistent, suggesting it is possible too, but also may not be a main effect for CFI measures.

Mean CFI, ITFI, and FR estimates are further biased and shortened by non-random sampling of multi-scarred trees. Van Horne and Fulé (2006) found a statistical difference, using 95% confidence intervals, between mean CFI for an individual-tree targeted sample and a large census. Comparison of a random sample and a targeted sample, each of 40 trees, shows that mean CFI in the targeted sample was 79.1% (2.23/2.82) of the mean CFI in the random sample for all fires, 98.4% (3.00/3.05) for mean CFI-10%-scarred, and 86.9% (5.43/6.25) for mean CFI-25%-scarred. Farris et al. (2013) re-analyzed the Van Horne and Fulé (2006) dataset and added two other datasets, which together showed targeted samples had a mean CFI-all fires that was 78.9-112.5%, a mean CFI-10% scarred that was 93.5-131.4%, and a mean CFI-25% scarred that was 80.0-96.1%, of the corresponding mean CFI from a probabilistic sample. In Brown et al. (2008), a target-supplemented sample (their Figure 4d) had a mean CFI that was 88.9% (24/27) of that from a systematic plot sample (their Figure 4c). ITFI and FR estimates from recorders are similarly affected. Van Horne and Fulé (2006) found that mean ITFI in a targeted sample was 83.3% of mean ITFI in a random sample. Everett (2003) sampled fire-scarred trees using a grid at two sites and chose the closest fire-scarred tree, thus a probabilistic sample without targeting multi-scarred trees. No comparable estimate from non-random sampling and a targeted sample was made, but Everett's estimated ITFIs were in the 3rd and 4th quartiles of the distribution of estimated ITFIs in the 96-case calibration dataset, consistent with the possibility that ITFIs were long because of lack of targeting.

Farris et al. (2013) showed that individual-tree targeting and non-random sampling even led to ratio-based estimates of FR at three sites that were reduced to 85.5%, 88.3%, and 94.8% of FR estimates from equal-sized probabilistic samples. As suggested earlier, this may be because places with long fire intervals that are real are omitted. These omissions may be places that particular fires did not burn, thus fire size for those fire years is inflated, leading to FR estimates that are too short.

Another impact of individual-tree targeting is reduced completeness of the fire record and over-representation by small, low-severity fires. Fiegener (2002) found that targeting trees with ≥ 5 scars reduced detected fires from 76 to 68 (to about 89.5%), but reduced detection of larger fires to 77%, thus increasing the proportion of small fires in the sample. Baker and Ehle (2003) also found that targeting multi-scarred trees identified and emphasized more small, low-severity fires, including one-tree fires that are another central source of bias in CFI estimates (Baker and Ehle 2001). A non-targeted sample did as well or better at identifying large, low-severity and mixed-severity fires (Baker and Ehle 2003). Also, 18% of 60 total fires and 30% of the most ancient fires (pre-1700), including a significant high-severity fire, found in a non-targeted sample would have been missed if only trees with ≥4 scars were sampled (Baker and Ehle 2003). Targeting multi-scarred trees thus leads to an incomplete fire record, missing significant fires, and a bias toward small fires that produce CFI and ITFI estimates that are too short.

A related type of individual-tree targeting focuses only on "recorder" trees with at least one previous fire scar (thus ≥2 fire scars), which are thought to preferentially record fires, leading to a more complete fire record. To have increased the probability of receiving a subsequent scar, these trees had to have been effectively open, with a scar lacking bark, at the time of the next fire. Previously scarred trees do have a much higher probability of receiving a new scar than do unscarred trees (Baker and Dugan 2013). However, they are much less common than unscarred trees, and unscarred trees appear to typically be scarred at a sufficient rate in a fire to outnumber scars on recorder trees. For example, in a single fire, 73% of scarred trees were first scars and only 27% were recorders that had a previous scar (Stephens et al. 2010), suggesting previously scarred trees were poorer recorders of the fire, in terms of number of scars per unit area, even though scarred at a higher rate. In a larger Rocky Mountain National Park (RMNP) study (Baker and Ehle 2003), for 24 fires that showed up both as first scars and on recorders, 62% of the scars documenting these 24 fires were not on recorders, while 38% were on recorders, a significant difference ($\chi^2 = 4.76$, $p = 0.029$) and lower rate per unit area for recorders, just as in the Stephens et al. study. Moreover, we found 60 total fires, and 32% of these fires showed up only as first scars while 28% of the 60 fires showed up only on recorders, suggesting neither source alone provides a complete fire history. However, there was not much difference in the ability of more numerous unscarred and less numerous recorder trees to record complete histories of fire. Moreover, recorders have the same additional biases, as estimators of PMFI/FR, as do other multi-scarred trees, as reviewed above.

Targeting open-scarred trees often aims at trees with a cat-face or deep semicircular wound, which typically also means they are multi-scarred trees and qualify as recorders. In a study of a single fire in a California Sequoia grove, 68% of open-scarred trees were scarred in a 1797 fire, but only 20% of intact trees were scarred (Kilgore and Taylor 1979). Across many fires, in a California study, a significantly greater mean fraction (0.22) of oaks with open scars at the time of a fire had scars from the fire than did intact trees (0.09), but twice as many intact trees on the sites had scars since there were 5.5 times as many intact trees as trees with open scars (McClaran 1988). This pattern is similar to that of recorders. Mean CFI did not differ between open-scarred and intact trees at one site, but open-scarred trees had 27% fewer fire dates (McClaran 1988). Thus, targeting open-scarred trees thought to be better recorders of fires also leads to omission of fires and the other biases of multi-scarred trees.

Species targeting focuses on particular tree species thought to be better recorders of fire. For example, one might obtain fire scars from ponderosa pine trees on the edge of piñon-juniper woodlands, because the ponderosa are thought to have a better record, from a higher SF (e.g., Miller and Rose 1999). However, fires that burned the ponderosa likely did not penetrate into the woodlands much, if at all (Huffman et al. 2008), thus the apparent difference in SF may reflect real differences in burning rates. To

avoid a targeting effect from assuming that the tree species with more scars has a more complete record, data can be acquired from piñon-juniper woodlands and adjusted for their lower scarring fraction. This is what the ATFI method allows, a separate SF for differing trees on the same site (Kou and Baker 2006a).

Individual-tree targeting of older trees for sampling occurs because older trees have a potentially longer record (Farris et al. 2013). This type of targeting may also occur if trees with multiple scars are targeted, since trees generally must get older before they have multiple scars. By definition, individual trees with long fire-scar records have a history of only low-severity fires at that tree, thus a targeted sample of only old trees is certain to indicate a long history of low-severity fire. When fire is moderate- to high-severity, evidence of fire severity on surviving older trees underestimates fire severity in the stand (Hessburg et al. 2007). A targeted sample of old trees in a landscape with trees of other ages thus provides strongly biased evidence about the fire severities that affected the stand.

Targeting sampling areas in landscapes

Targeting particular landscapes or parts of landscapes also leads to bias, generally toward CFI and ITFI estimates that are too short relative to PMFI/FR, since the methods of individual-tree targeting are also used at the landscape scale. Researchers seeking to reconstruct pre-EuroAmerican fire regimes may select parts of landscapes with concentrations of multi-scarred trees, recorders, open-scarred trees or catfaces, and old trees or old-growth forests. In almost 1/3 of the cases where targeting or lack of it was reported, researchers located plots specifically in these areas and in about 10% of cases researchers chose study areas with these concentrations (Table A). These plot locations and study areas may contain long fire records and many fire scars, and are attractive to researchers seeking long fire records (Farris et al. 2013). However, these parts of landscapes also are forests that had a predominance of low-severity fire and little to no mixed- or high-severity fire for hundreds of years, as most trees would otherwise be younger. As explained in the main text, researchers may target areas with abundant fire scars and omit or reduce sampling in areas that lack or have few scars, then also may inappropriately assume that fire history in areas with abundant fire scars also applies to areas with few or no fire scars.

In contrast, probabilistic sampling areas, particularly if appropriately small (e.g., 1 ha) may commonly lack scarred trees or have few. Heyerdahl (1997) sampled using plots located in a grid, thus without targeting sampling areas in landscapes, and found that scarred trees were lacking in more than half the plots at three study sites. These areas could in part have had few scars because of a low scarring fraction, but could also have been areas that really did not burn for a long period. If the latter, then omitting these long intervals, that are real, would bias results toward underestimating fire severity and bias estimated rates of low-severity fire toward shorter intervals. This kind of targeting is not clearly rejected by supporters of targeting (Farris et al. 2013 p. 1030), although they encourage "...clearly defining the inference space, not extrapolating to unrepresentative areas..." This kind of targeting did clearly include extrapolating to unrepresentative areas in past fire histories that are the subject of this paper. I am not singling out particular authors, as most used sampling methods that were common practice at the time, largely aimed at finding and sampling the best evidence (Farris et al. 2013).

However, targeting of old forests, that inherently have a history of low-severity fire, likely explains the unexpected findings of landscape analyses of fire history that did not use targeting. When an objective, large sample (303,156 ha) of historical dry forests was studied in the Pacific Northwest using early aerial photography, middle-aged forests resulting from mixed- and high-severity fires were found to have dominated historical landscapes and old, park-like forests, exclusively with low-severity fire, were found to have been comparatively uncommon (Hessburg et al. 2007 p. 7): "Moreover, old, park-like or similar ponderosa pine stand structures did not dominate the landscapes, and this was particularly perplexing because this was to be the signature outcome of frequent low severity fires." Similarly, spatially extensive reconstruction across landscapes using the early land surveys, found evidence of abundant denser and younger forests from mixed- and high-severity fire across dry forests in northern Arizona, the Colorado Front Range, and the Blue Mountains in Oregon, where previous fire-history

studies had found predominantly low-severity fire and open, low-density old forests (Williams and Baker 2012). Finally, stand age data from the Forest Inventory and Analysis Program also showed that young and middle-aged forests, not park-like old forests, were most common historically in ponderosa pine and mixed-conifer forests across relatively undisturbed parts of the western USA (Odion et al. 2014). These studies with probabilistic sampling at the landscape scale show that targeting parts of landscapes containing old forests with abundant fire scars led to very substantial over-estimation of the historical extent of low-density old forests that predominantly had low-severity fire.

Unstudied fire severity in dry forests also inflates low-severity fire rates

Estimates from CFIs, ITFIs, and FRs likely often included all fire severities, not just low severity, and the low-severity rates alone are thus likely longer. Fire severity has been relatively infrequently studied in dry forests. Baker and Ehle (2003) found that only about 25% of fire-scar studies also collected the age-structure data needed to determine whether higher-severity fires occurred historically. Of the 335 cases in the merged dataset with data, 254 (74.3%) did not study fire severity, 80 (23.4%) did study fire severity, and 8 (2.3%) did not explain whether they studied fire severity. Most studies that did analyze fire severity did not distinguish fire severities when they reported fire rates (e.g., Taylor and Skinner 1998). Where fire severity was studied, some mixed- or high-severity fires were nearly always found, but few studies estimated PMFI or FR for the higher-severity fires. Thus, most fire-history studies provide estimates of rates for all fires combined, including low, moderate- and high-severity fires.

The potential effect of combined fire severities on estimated rates for low-severity fire can be illustrated by subtracting, using partitioning (Baker 2009), reported rates of moderate- to high-severity fire from rates of low-severity fire. Odion et al. (2014) reported historical rates of combined moderate- to high-severity fire ranged from 115-128 years in the eastern Cascades of Oregon to 319 years on the Mogollon Plateau. I used the full range of 115-319 years to remove the moderate- to high-severity component, and found that 10-year combined PMFI/FRs would have a 10.3-11.0 low-severity component, but 50-year combined PMFI/FRs would have a 59.3-88.5-year low-severity PMFI/FR after removing the moderate- to high-severity component (Table B). I did not apply an adjustment, for this fire-severity issue, to estimated PMFI/FRs because the adjustments are imprecise and have a large range, and because sites where fire severity was unstudied did not necessarily have higher-severity fires. Nonetheless, this finding illustrates the limitation of unstudied fire severity, and shows that many estimates of low-severity PMFI/FR are likely low estimates.

Table B. Partitioning combined fire rotations (FR) into components for low- versus moderate and high-severity fire for three example levels of combined fire rotations.

	10-year combined-severity PMFI/FR	25-year combined-severity PMFI/FR	50-year combined-severity PMFI/FR
a. Combined annual probability of fire (1/FR)	0.10000	0.04000	0.02000
UPPER LIMIT OF LOW-SEVERITY RANGE			
b. Annual probability of fire for moderate-high component of 115 years†	0.00870	0.00870	0.00870
c. Annual probability of fire for low-severity component, from a - b.	0.09130	0.03130	0.01130
d. Net fire rotation for low-severity component, from 1 / c.	10.95 years	31.95 years	88.50 years
LOWER LIMIT OF LOW-SEVERITY RANGE			

e. Annual probability of fire for moderate-high component of 319 years†	0.00313	0.00313	0.00313
f. Annual probability of fire for low-severity component, from a - e.	0.09687	0.03687	0.01687
g. Net fire rotation for low-severity component, from 1 / g.	10.32 years	27.12 years	59.28 years
NET ESTIMATED LOW-SEVERITY RANGE	10.32-10.95 years	27.12-31.95 years	59.28-88.50 years

Notes

† The 115-319 year range for moderate- to high-severity fire rotation in dry forests is from Odion et al. (2014)

Literature Cited

Arno, S. F., and T. D. Petersen. 1983. Variation in estimates of fire intervals: a closer look at fire history on the Bitterroot National Forest. USDA Forest Service Research Paper INT-301, Intermountain Forest and Range Experiment Station, Ogden, Utah.

Baker, W. L. 2009. Fire ecology in Rocky Mountain landscapes. Island Press, Washington, D.C..

Baker, W. L., and A. J. Dugan. 2013. Fire-history implications of fire scarring. Canadian Journal of Forest Research 43:951-962.

Baker, W. L., and D. Ehle. 2001. Uncertainty in surface-fire history: the case of ponderosa pine forests in the western United States. Canadian Journal of Forest Research 31:1205-1226.

Baker, W. L., and D. S. Ehle. 2003. Uncertainty in fire history and restoration of ponderosa pine forests in the western United States. Pages 319-333 *in* P. N. Omi and L. A. Joyce editors. Fire, fuel treatments, and ecological restoration: Conference proceedings [April 16-18, 2002--Fort Collins, CO]. USDA Forest Service Proceedings RMRS-P-29, Rocky Mountain Research Station, Fort Collins, CO.

Brown, P. M., C. L. Wienk, and A. J. Symstad. 2008. Fire and forest history at Mount Rushmore. Ecological Applications 18:1984-1999.

Collins, B. M., and S. L. Stephens. 2007. Fire scarring patterns in Sierra Nevada wilderness areas burned by multiple wildland fire use fires. Fire Ecology Special Issue 3:53-67.

Dugan, A. J., and W. L. Baker. 2014. Modern calibration and historical testing of small-area, fire-interval reconstruction methods. International Journal of Wildland Fire 23:58-68.

Everett, R. G. 2003. Grid-based fire-scar dendrochronology and vegetation sampling in the mixed-conifer forests of the San Bernardino and San Jacinto Mountains of southern California. PhD dissertation, University of California, Riverside, California.

Farris, C. A., C. H. Baisan, D. A. Falk, M. L. Van Horne, and Fulé. 2013. A comparison of targeted and systematic fire-scar sampling for estimating historical fire frequency in south-western ponderosa pine forests. International Journal of Wildland Fire 22:1021-1033.

Farris, C. A., C. H. Baisan, D. A. Falk, S. R. Yool, and T. W. Swetnam. 2010. Spatial and temporal corroboration of a fire-scar-based fire history in a frequently burned ponderosa pine forest. Ecological Applications 20:1598-1614.

Fiegener, R. P. 2002. The influence of sampling intensity on the fire history of the Teakettle Experimental Forest, Sierra Nevada, California: small fire detection, the composite fire chronology, and fire interval calculation. University of California, Davis.

Hessburg, P. F., M. James, and R. B. Salter. 2007. Re-examining fire severity relations in pre-management era mixed conifer forests: inferences from landscape patterns of forest structure. Landscape Ecology 22:5-24.

Heyerdahl, E. K. 1997. Spatial and temporal variation in historical fire regimes of the Blue Mountains,

Oregon and Washington: the influence of climate. Ph.D. dissertation. University of Washington, Seattle.
Huffman, D. W., P. Z. Fulé, K. M. Pearson, and J. E. Crouse. 2008. Fire history of pinyon-juniper woodlands at upper ecotones with ponderosa pine forests in Arizona and New Mexico. Canadian Journal of Forest Research 38:2097-2108.
Johnson, E. A., and S. L. Gutsell. 1994. Fire frequency models, methods and interpretations. Advances in Ecological Research 25:239-287.
Kilgore, B. M., and D. Taylor. 1979. Fire history of a sequoia-mixed conifer forest. Ecology 60:129-142.
Kou, X., and W. L. Baker. 2006a. Accurate estimation of mean fire interval for managing fire. International Journal of Wildland Fire 15:489-495.
Kou, X., and W. L. Baker. 2006b. A landscape model quantifies error in reconstructing fire history from scars. Landscape Ecology 21:735-745.
Lorimer, C. G. 1985. Methodological considerations in the analysis of forest disturbance history. Canadian Journal of Forest Research 15:200-213.
McClaran, M. P. 1988. Comparison of fire history estimates between open-scarred and intact Quercus douglasii. American Midland Naturalist 120:432-435.
Miller, R. F., and J. A. Rose. 1999. Fire history and western juniper encroachment in sagebrush steppe. Journal of Range Management 52:550-559.
Moritz, M. A., T. J. Moody, L. J. Miles, M. M. Smith, and P. De Valpine. 2009. The fire frequency analysis branch of the pyrostatistics tree: sampling decisions and censoring in fire interval data. Environmental and Ecological Statistics 16:271-289.
Odion, D. C., C. T. Hanson, A. Arsenault, W. L. Baker, D. A. DellaSala, R. L. Hutto, W. Klenner, M. A. Moritz, R. L. Sherriff, T. T. Veblen, and M. A. Williams. 2014. Examining historical and current mixed-severity fire regimes in ponderosa pine and mixed-conifer forests of western North America. PloS One 9(2):e87852.
Parsons, R. A., E. K. Heyerdahl, R. E. Keane, B. Dorner, and J. Fall. 2007. Assessing accuracy of point fire intervals across landscapes with simulation modelling. Canadian Journal of Forest Research 37:1605-1614.
Polakow, D. A., and T. P. Dunne. 1999. Modelling fire-return interval *T*: stochasticity and censoring in the two-parameter Weibull model. Ecological Modelling 121:79-102.
Stephens, S. L., D. L. Fry, B. M. Collins, C. N. Skinner, E. Franco-Vizcaino, and T. J. Freed. 2010. Fire-scar formation in Jeffrey pine-mixed conifer forests in the Sierra San Pedro Mártir, Mexico. Canadian Journal of Forest Research 40:1497-1505.
Stephens, S. L., C. N. Skinner, and S. J. Gill. 2003. Dendrochronology-based fire history of Jeffrey pine-mixed conifer forests in the Sierra San Pedro Martir, Mexico. Canadian Journal of Forest Research 33:1090-1101.
Taylor, A. H., and C. N. Skinner. 1998. Fire history and landscape dynamics in a late-successional reserve, Klamath Mountains, California, USA. Forest Ecology and Management 111:285-301.
Van Horne, M. L., and P. Z. Fulé. 2006. Comparing methods of reconstructing fire history using fire scars in a southwestern United States ponderosa pine forest. Canadian Journal of Forest Research 36:855-867.
Williams, M. A., and W. L. Baker. 2012. Spatially extensive reconstructions show variable-severity fire and heterogeneous structure in historical western United States dry forests. Global Ecology and Biogeography 21:1042-1052.
